# Sample size estimation in clinical trials using ventilator-free days as the primary outcome: a systematic review

**DOI:** 10.1186/s13054-023-04562-y

**Published:** 2023-08-01

**Authors:** Laurent Renard Triché, Emmanuel Futier, Manuela De Carvalho, Nathalie Piñol-Domenech, Laëtitia Bodet-Contentin, Matthieu Jabaudon, Bruno Pereira

**Affiliations:** 1 Department of Perioperative Medicine, CHU Clermont-Ferrand, 58 Rue Montalembert, 63000 Clermont-Ferrand, France; 2 iGReD, CNRS, INSERM, Université Clermont Auvergne, Clermont-Ferrand, France; 3 Université Clermont Auvergne, Health Library, Clermont-Ferrand, France; 4 Medical Intensive Care Unit, CHRU de Tours, Tours, France; 5 INSERM, SPHERE, UMR1246, Université de Tours et Nantes, Tours et Nantes, France; 6 Biostatistics Unit, Department of Clinical Research, and Innovation (DRCI), CHU Clermont-Ferrand, Clermont-Ferrand, France

**Keywords:** Respiration, artificial, Respiratory insufficiency, Time factors, Treatment outcome, Ventilators, mechanical

## Abstract

**Background:**

Ventilator-free days (VFDs) are a composite endpoint increasingly used as the primary outcome in critical care trials. However, because of the skewed distribution and competitive risk between components, sample size estimation remains challenging. This systematic review was conducted to systematically assess whether the sample size was congruent, as calculated to evaluate VFDs in trials, with VFDs’ distribution and the impact of alternative methods on sample size estimation.

**Methods:**

A systematic literature search was conducted within the PubMed and Embase databases for randomized clinical trials in adults with VFDs as the primary outcome until December 2021. We focused on peer-reviewed journals with 2021 impact factors greater than five. After reviewing definitions of VFDs, we extracted the sample size and methods used for its estimation. The data were collected by two independent investigators and recorded in a standardized, pilot-tested forms tool. Sample sizes were calculated using alternative statistical approaches, and risks of bias were assessed with the Cochrane risk-of-bias tool.

**Results:**

Of the 26 clinical trials included, 19 (73%) raised “some concerns” when assessing risks of bias. Twenty-four (92%) trials were two-arm superiority trials, and 23 (89%) were conducted at multiple sites. Almost all the trials (96%) were unable to consider the unique distribution of VFDs and death as a competitive risk. Moreover, significant heterogeneity was found in the definitions of VFDs, especially regarding varying start time and type of respiratory support. Methods for sample size estimation were also heterogeneous, and simple models, such as the Mann–Whitney–Wilcoxon rank-sum test, were used in 14 (54%) trials. Finally, the sample sizes calculated varied by a factor of 1.6 to 17.4.

**Conclusions:**

A standardized definition and methodology for VFDs, including the use of a core outcome set, seems to be required. Indeed, this could facilitate the interpretation of findings in clinical trials, as well as their construction, especially the sample size estimation which is a trade-off between cost, ethics, and statistical power.

*Systematic review registration* PROSPERO ID: CRD42021282304. Registered 15 December 2021 (https://www.crd.york.ac.uk/prospero/display_record.php?ID=CRD42021282304).

**Supplementary Information:**

The online version contains supplementary material available at 10.1186/s13054-023-04562-y.

## Background

Between a quarter and half of the patients admitted to the intensive care unit will present with respiratory failure, requiring invasive mechanical ventilation [1]. These patients are at risk of complications, such as ventilator-associated pneumonia and death, with related health-care costs [2, 3].

Mortality is a robust endpoint that has long been used in studies [4]. However, since the improvement of therapeutics, mortality has decreased [5], and the sample size needed to show a clinically relevant difference in mortality has also increased. Hence, most published randomized clinical trials (RCTs) that aim to reduce mortality have produced negative results [6, 7]. For this reason, other outcomes have been developed, such as ventilator-free days (VFDs), which are increasingly used in critical care RCTs [8]. First proposed in 1994 [9], VFDs were developed in studies focusing on acute respiratory distress syndrome. The number of VFDs was defined as the number of days from the last day of mechanical ventilation to day 28. If a patient died during the first 28 days, their number of VFDs is equal to zero. This composite outcome measure (i.e., combining survival and the duration of ventilation) is more appropriate than only the duration of ventilation because the latter disregards the mortality rate [10].

In clinical research, it is not feasible, for most studies, to study the whole population [11]. We therefore need to determine the sample size, which can be imprecise and difficult. Indeed, it represents a trade-off between cost effectiveness (i.e., in terms of time and resource), ethical concerns (e.g., an oversized experiment would result in exposure of an unnecessary number of subjects) and statistical power (i.e., a small sample size could make the study underpowered to show a clinically meaningful difference, if any, and to detect a potentially effective treatment) [12]. Calculating this sample size involves the employment of formulae designed to obtain significant results in studies that compare several groups based on the primary endpoint. The test chosen to analyze the primary endpoint will depend on its distribution and is part of the sample size estimation [13]. VFDs do not follow a Gaussian distribution [14]; therefore, we cannot use parametric tests. Indeed, the distribution is skewed with inflations, especially 0 s, and represents a rather time-dependent event. In their last review, Yehya et al. [8] recommended using competing risk regression, such as the Fine and Gray competing risk regression [15], which considers extubation success as the event of interest and death as the competing risk.

There appears to be a number of inconsistencies in the definitions and methodologies used for VFDs in the literature [8, 16]. As a result, we conducted a systematic review of RCTs using VFDs as the primary outcome to evaluate them. Hence, our principal objective was to investigate whether the sample size estimation of VFDs was congruent with their true distribution. Indeed, incorrect sample size estimation may lead to additional costs, expose an unnecessary number of subjects or decrease the power of a study. Our secondary objectives were to review the definitions of VFDs and to evaluate different statistical approaches to their estimation.

## Methods

### Search strategy, study selection and inclusion criteria

We searched through two databases (MEDLINE and Embase) using a combination of keywords. The last literature search was done on December 31, 2021. We only focused on RCTs with VFDs as the primary outcome in peer-reviewed journals with 2021 impact factors greater than five. The complete list of search terms is available in the online data supplement (Additional file 1: Appendix 1). Two investigators (LRT and MJ) independently screened the titles and abstracts of the search results. The full text of all potentially eligible studies was retrieved and reviewed for eligibility. First, we removed the duplicates between the databases. Then, we excluded all trials that were not RCTs in adults and those with the primary endpoint that was not VFDs. A narrative synthesis supporting the Preferred Reporting Items for Systematic Reviews and Meta-Analyses (PRISMA) flow diagram and the PRISMA 2020 Checklist [17] was included as part of this systematic review. The study protocol was registered with the International Prospective Register of Systematic Reviews (PROSPERO) in December 2021 (ID: CRD42021282304) [18].

### Data extraction

Data were extracted independently by two investigators (LRT and MJ or BP) and collected into standardized forms using Research Electronic Data Capture tools [19, 20]. Data were cross-checked; any disagreements were resolved first by consensus, and if one or several disagreements persisted, a third investigator (BP or MJ) was involved. For each selected article, we recorded several items, as detailed in the online data supplement (Additional file 1: Appendix 2).

### Risk of bias

To assess the risk of bias in each study, we used Version 2 of the Cochrane risk-of-bias tool (RoB2) for RCTs [21]. The studies were assessed using five fixed domains, as outlined in RoB2. Each study was classified by two investigators (LRT and MJ) as having “low risk,” “some concerns,” or “high risk.”

### Outcomes

Our primary outcome was the sample size, as estimated in RCTs evaluating VFDs as their primary outcome. First, we extracted the sample sizes estimated and observed among the trials. Secondly, because there is heterogeneity in the tests used for this outcome, we simulated other sample sizes through alternative statistical approaches. Our secondary outcomes were to review the definitions of VFDs, mortality rates, statistical methods and VFDs’ distributions among selected trials.

### Statistical analysis

The different statistical approaches were as follows: the Student *t*-test and the Mann–Whitney–Wilcoxon rank-sum test because these are standard tests used in several studies; the Mann–Whitney–Wilcoxon rank-sum test using the Noether formula to compare if the result differs from the previous one; the Cox regression because VFDs are considered by some to be a time-dependent event; the zero-inflated negative binomial regression because VFDs involve a zero-inflation; and finally, the Fine and Gray regression because the VFDs involve death as a competitive risk. The corresponding formulae are available in the online data supplement (Additional file 1: Appendix 3). All statistical analyses were performed using R Core Version 4.2 [22]. All packages used are listed in the online data supplement (Additional file 1: Appendix 4).

## Results

### Study selection and characteristics

We identified 425 studies from 2004 to 2021. After removing duplicates, we assessed 269 studies. One hundred and thirty-six non-randomized studies were excluded, as well as two animal studies and 29 pediatric studies. We then excluded 76 studies in which the primary outcome was not VFDs. Twenty-six studies were finally included in our systematic review [23–48] (Additional file 1: Fig. S1). These were 24 (92%) superiority studies comparing two groups (three for the two remaining) among several centers (median [IQR], 23 [8﻿–42]) for 22 (85%) trials. An interim analysis was performed in 16 (58%) trials. Ten (39%) of the selected studies had to be stopped early. Finally, the patient populations included in these studies were heterogeneous, with a third having acute respiratory distress syndrome (see Table 1 and Additional file 1: Table S1).

**Table 1:** Characteristics of the included studies

Variable	*n*/*N* (%) or median [IQR]
Year of publication	2014 [2011–2020]
Journal	
AIM	1/26 (4)
AJRCCM	4/26 (15)
CCM	6/26 (23)
ICM	4/26 (15)
JAMA	7/26 (27)
LRM	1/26 (4)
NEJM	3/26 (12)
Number of group—ratio (E/C)*	
2–1:1	23/26 (88)
2–4:1	1/26 (4)
3–1:1:1	2/26 (8)
Study type	
Superiority	24/26 (92)
Noninferiority	2/26 (8)
Number of center if multicentric^†^	23 [﻿8–42]
Interim analysis	16/26 (62)
Stopped earlier than expected	10/26 (39)
Population	
ARDS or ALI	14/26 (54)
COVID-19	3/26 (12)
Overall RoB2^‡^	
Low risk	3/26 (12)
Some concerns	19/26 (73)
High risk	4/26 (15)

*AIM* Annals of internal medicine, *AJRCCM* American Journal of Respiratory and Critical Care Medicine, *ALI* acute lung injury, *ARDS* acute respiratory distress syndrome, *CCM* Critical Care Medicine, *ICM* Intensive Care Medicine, *IQR* interquartile range, *JAMA* Journal of the American Medical Association, *LRM* The Lancet Respiratory Medicine, *n* number of study, *N* total number of studies, *NEJM* The New England Journal of Medicine

*The allocation ratio (E for experimental group and C for control group)

^†^There were 22 (85%) studies with more than one center

^‡^The overall risk-of-bias via RoB2 (Revised Cochrane risk-of bias tools for randomized trials) [21]

### Risk of bias and disagreements

Using the Excel spreadsheet provided by the RoB2 tool, we assessed the risk of bias, as summarized in Additional file 1: Table S2. Nineteen (73%) of the studies were assessed as having “some concerns,” mainly related to the randomization process and deviations from the intended interventions.

Among all the collected items, the median [IQR] number of disagreements between the two reviewers was 1 [0–2] out of the 26 selected studies, for a total of 23 disagreements out of 769 items (3%). All disagreements were resolved by consensus.

### Sample size estimation

We extracted the estimated and observed sample sizes reported in the selected studies. We subsequently estimated the sample size with parameters (e.g., risk, power, mean difference) reported in two ways: using the expected parameters displayed in the Material and Methods section or the observed parameters displayed in the Results section.

First, we reported the expected parameters proposed by the authors for the sample size estimation in the Methods sections of the selected studies (see Table 2 and Additional file 1: Table S3). The absolute mean difference in VFDs ranged from 0.5 to 7.0. In one noninferiority study [30], the authors considered 1.6 to be the noninferiority margin, whereas in one superiority study [29], the authors considered 1 to be the superiority margin. These expected mean differences were only justified in eight (31%) studies. The standard deviation was only reported in 23% of studies, but when it was available, it was heterogenous (median [IQR], 10.0 [6.8–10.5]) (Additional file 1: Fig. S2). Mortality was considered in one study only [26], in which Markov chains considered the probabilities of death, getting off ventilation alive, and receiving ventilation. Finally, the expected dropout rate was quite diverse among studies (0–25%).

**Table 2:** Parameters reported for the sample size estimation of ventilator-free days

Variable	*n*/*N* (%) or median [IQR]	NA (%)
Expected VFDs		
Control group	14.00 [11.90–16.70]	10 (38)
Experimental group	16.25 [14.88–19.77]	10 (38)
Mean difference	2.60 [2.00–7.00]	3 (12)
Standard deviation	10.00 [6.75–10.53]	6 (23)
VFDs distribution		13 (50)
Asymmetric	3/26 (11)	
Bimodal	1/26 (4)	
Normal	2/26 (8)	
Not normal	5/26 (19)	
Zero-inflated distribution	2/26 (8)	
Correction if distribution was not considered normal		15 (57)
+ 15%	3/26 (12)	
Markov chains	1/26 (4)	
Median comparison	1/26 (4)	
No correction	2/26 (8)	
Nonparametric test	4/26 (15)	
Statistical model		5 (19)
ANOVA	5/26 (19)	
Kruskal–Wallis	1/26 (4)	
Student *t*-test	2/26 (8)	
Mann–Whitney–Wilcoxon rank-sum test	8/26 (30)	
Quantile regression	1/26 (4)	
Cox regression	1/26 (4)	
GAMLSS	1/26 (4)	
GLM	2/26 (8)	
Risk	5 [5–5]	0 (0)
Power (%)	80 [80–80]	1 (4)
Two-tailed test	21 (81)	1 (4)
Dropout rate expected	3.00 [0.00–9.00]	0 (0)
Mortality considered for sample size estimation	1/26 (4)	0 (0)

*ANOVA* analysis of variance, *GAMLSS* generalized additive model for location scale and shape, *GLM* generalized linear model, *IQR* interquartile range, *n* number of study, *N* total number of studies, *NA* not available, *VFDs* ventilator-free days

**Fig. 1:**
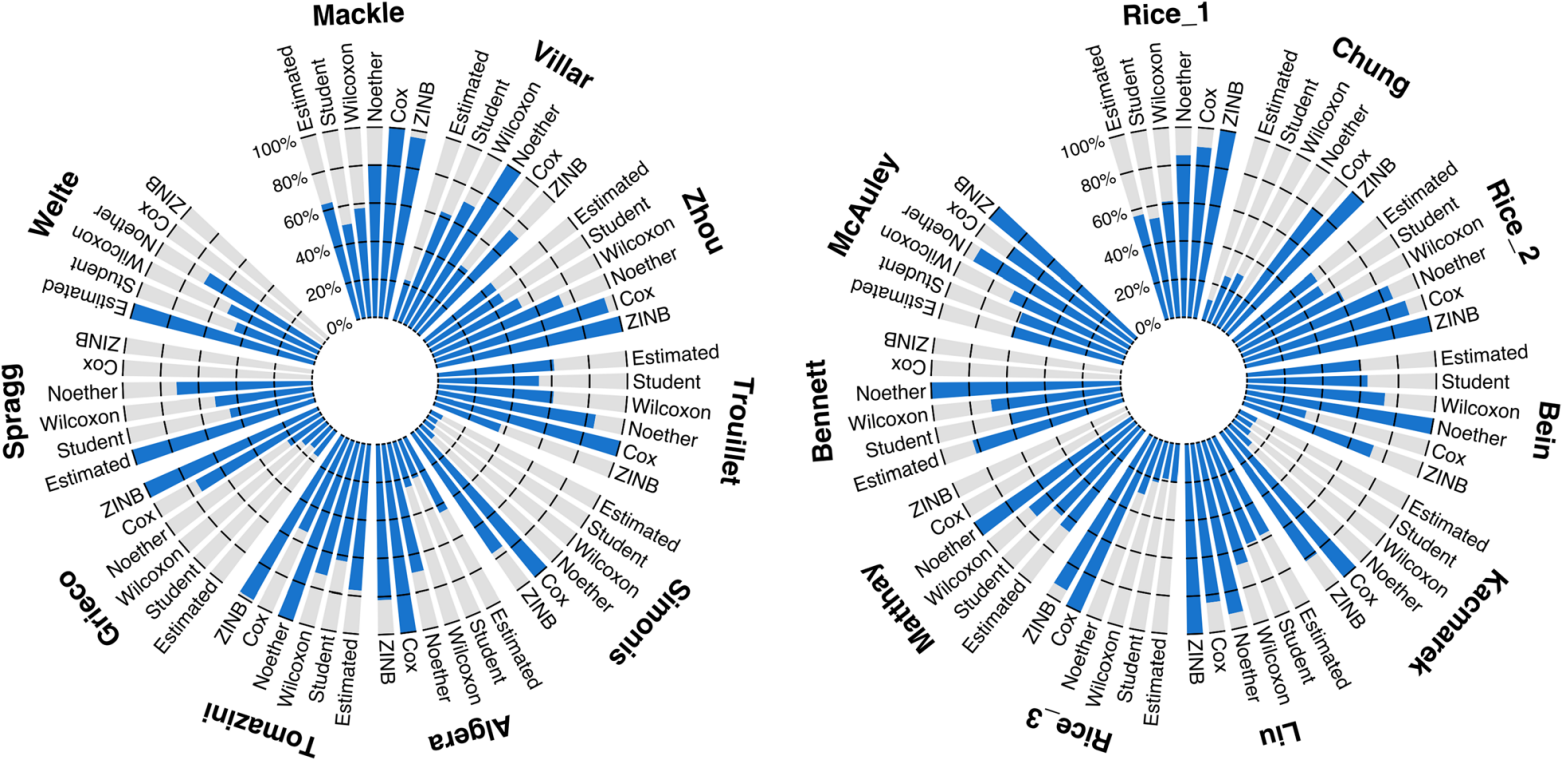
Sample size estimation as reported in each trial and computed according to different alternative tests. For each study, sample size estimation is plotted (in blue) against the highest value among the sample size estimated in the study and five different tests: the Student *t*-test, the Mann–Whitney–Wilcoxon rank-sum test, the Mann–Whitney–Wilcoxon rank-sum test using the Noether formula, Cox regression and zero-inflated negative binomial (ZINB) regression. When an estimation is missing, the whole length of the line is gray. The estimation was only possible for the following studies: Mackle [25]; Villar [26]; Zhou [27]; Trouillet [28]; Simonis [29]; Algera [30]; Tomazini [31]; Grieco [32]; Spragg [34]; Welte [35]; Rice_1 [36]; Chung [38]; Rice_2 [39]; Bein [40]; Kacmarek [42]; Liu [44]; Rice_3 [45]; Matthay [46]; Bennett [47]; and McAuley [48]

Using these parameters (i.e., mainly mean difference and standard deviation), we calculated, as reported in Additional file 1: Table S4, the different sample sizes resulting from different statistical tests: the Student *t*-test, the Mann–Whitney–Wilcoxon rank-sum test using the Noether formula or not, the Cox regression, the zero-inflated negative binomial (ZINB) regression, and the Fine and Gray regression. Several models could not be computed because of some expected parameters not being reported, such as the VFDs in the control group and their standard deviation. Moreover, it was not possible to estimate the sample size using the Fine and Gray regression because neither the probability of extubation nor the mortality incidence was reported. For estimations using Cox and ZINB regression, in most cases, the sample size was greater than with other models and slightly higher with Cox regression than with ZINB regression (see Fig. 1). The median [IQR] of the maximum variation factor between sample size estimations was 1.9 [1.7–3.5], with a maximum of 17.4.

Second, we reported the observed parameters needed to estimate the sample size (Additional file 1: Table S5). Standard deviations were slightly different from those estimated (absolute median difference [IQR], 4.5 [1.0–6.9]). Furthermore, the dropout rate observed was very low (0–2%).

Using these parameters, we calculated the different sample sizes using the same statistical tests as above (Additional file 1: Table S6). Sample sizes could not be estimated using the Fine and Gray regression model because, for some studies, VFDs and the mortality incidence had different timeframes. In addition, the incidence of extubation was never reported in the selected studies. We did not estimate the sample sizes in about half of the studies because the observed mean difference was too low (i.e., when the mean difference was less than 1). Indeed, conducting a study with such an effect size would appear irrelevant and clinically unrealistic.

Finally, because several data useful to estimate the sample size, especially for the Fine and Gray regression, were not reported; simulation was carried out from a previously published dataset by Bodet-Contentin et al. [49]. We estimated the sample size using this simulation and the same tests as above (Additional file 1: Table S7). Only the estimation using the Fine and Gray regression model provided a realistic sample size. However, this was more of a thought experiment because the effect size was low (mean difference = 0.46), and further simulation studies are warranted.

### Definitions of ventilator-free days

The definitions of VFDs across selected studies are reported in Table 3. Almost all studies counted whole days without support ventilation (92%) and calculated VFDs at day 28 after randomization. Other definitions were heterogeneous. The onset (i.e., the beginning of the period without support ventilation) was not the same across studies: 35% considered the onset at extubation and 38% at 48 h after extubation. If a patient was intubated again after a period of extubation, the count of VFDs started only after the last extubation event in 35% of the studies, and 27% of the studies summed the different periods during which the patient was extubated; the remaining studies did not specify this point. Finally, half of the studies did not mention the type of respiratory support (invasive or noninvasive) used to define VFDs.

**Table 3:** Definitions of ventilator-free days

Variable	*n*/*N* (%)
Onset	
No onset reported	9/26 (35)
> 4 h	1/26 (4)
> 24 h	3/26 (11)
> 48 h	10/26 (38)
> 72 h	1/26 (4)
Portion of day with no onset reported	2/26 (8)
Respiratory support	
Invasive ventilation	13/26 (50)
Tracheostomy	12/26 (46)
ECMO	2/26 (8)
NIV	6/26 (23)
HFNO	1/26 (4)
No detail reported	13/26 (50)
Reintubation	
Possible	7/26 (27)
Reset	9/26 (35)
No detail reported	10/26 (38)
Day when VFDs are defined	
28	24/26 (92)
30	1/26 (4)
60	1/26 (4)

*ECMO* extracorporeal membrane oxygenation, *HFNO* high-flow nasal oxygen, *n* number of study, *N* total number of studies, *NIV* noninvasive ventilation, *onset* time at which the patient is considered free of ventilation and start counting whole days except for two studies that considered portion of day, *VFDs* ventilator-free days

### Statistical methods to analyze ventilator-free days: distribution and statistical tests used

**Fig. 2:**
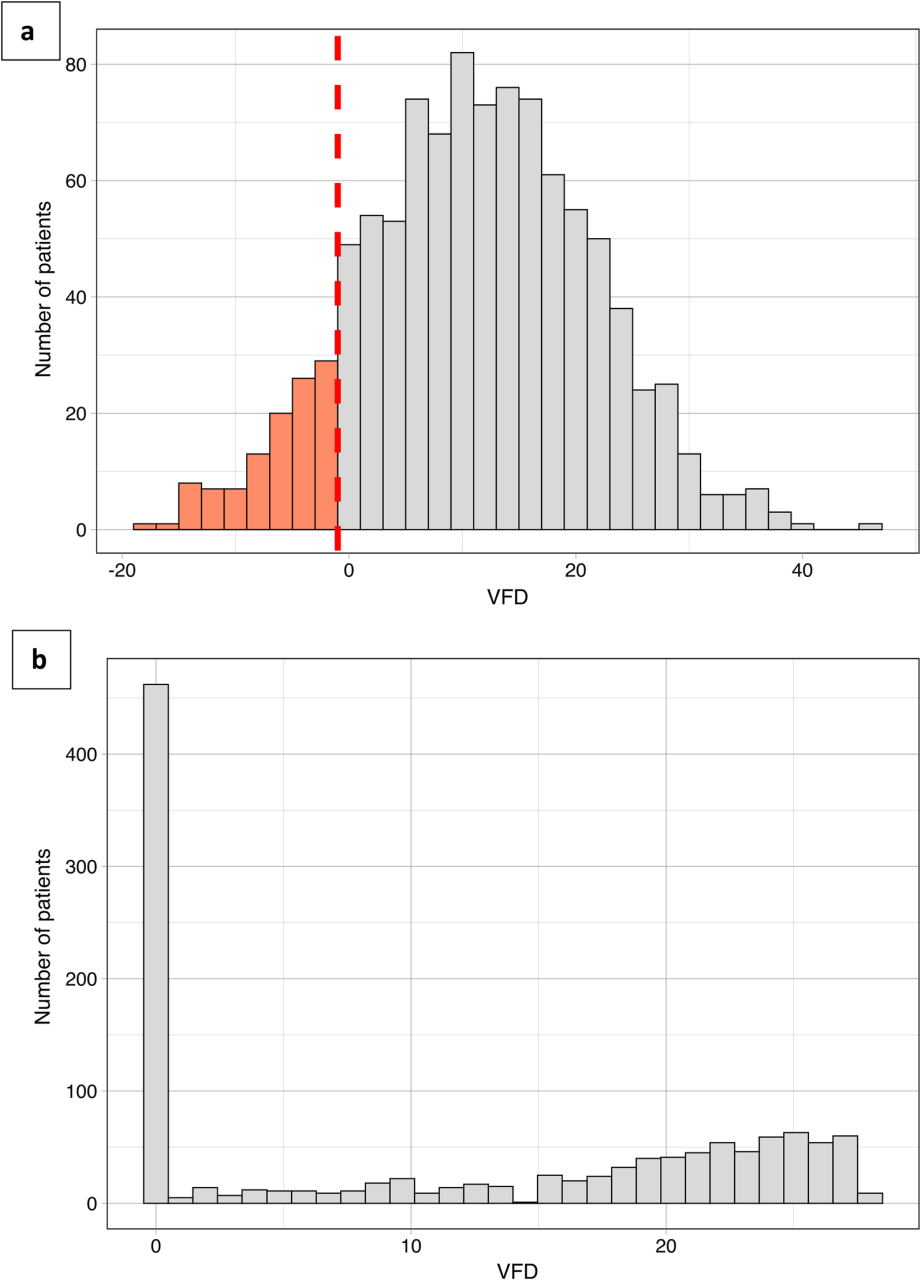
Distribution of ventilator-free days (VFDs) in selected trials. Histograms representing **a** a Gaussian distribution (mean of 11.7 days, standard deviation of 10.5) used in some studies for sample size estimation and **b** the empirical distribution of VFDs (mean of 11.7 days, standard deviation of 10.71 and median of 12.23, interquartile range 0.00–22.00) found in Jabaudon et al.’s meta-analysis [50]. The red bars correspond to the theoretical data that should be seen if the distribution were normal

The proper sample size estimation necessitates a correct estimation of the distribution of VFDs. Distributions of VFDs, as defined by the authors of the selected studies, are reported in Table 2 and Additional file 1: Table S3. Half of the studies did not explicitly state the type of assumed distribution, whereas the other half did not consider VFDs to be normally distributed. A more precise description was available for some studies, with 8% assuming a zero-inflated binomial distribution and 4% assuming a bimodal distribution. However, two recently published studies [32, 35] considered the number of VFDs to be normally distributed. We therefore simulated a normal distribution of VFDs with the parameters used in some trials included in our systematic review, which was not consistent with the empirical distribution of VFDs found in Jabaudon et al.’s meta-analysis [50] (see Fig. 2).

We also reported the statistical analysis methods used to assess VFDs in the selected studies (see Table 2 and Additional file 1: Table S3). About one-third used the Mann–Whitney–Wilcoxon rank-sum test, one-fifth did not specify the test used, and more than a third used parametric tests. A minority used complex models, such as generalized linear mixed models (GLMM) and generalized additive models for location, scale, and shape (GAMLSS). The main effect size reported was the absolute mean difference (in 69% of studies) (Additional file 1: Table S4). A significant result for VFDs was obtained only in a few studies (35%), but it was significant when a complex model was used (Additional file 1: Table S6).

### Other characteristics

The power ranged from 80% (for 73% of the studies) to 90%, and the α risk ranged from 2.5 to 10% (for one study [37]); however, the α risk was most frequently 5% (see Table 2 and Additional file 1: Table S3). The reported α risk was one-sided in four studies (two were noninferiority studies [30, 33], and two were superiority studies [35, 37]).

The most used timeframe for mortality was 28 days (Additional file 1: Table S5), corresponding to the same frame as for VFDs. The timeframe was reported in days in 92% of the studies; the remaining two used hospital or intensive care unit mortality. The incidence of death was quite different across the studies but similar within studies, with a median [IQR] of 24.9 days [19.2–30.9] for the control group and 24.3 days [19.8–30.7] for the experimental group.

Finally, only three (12%) trials planned multiple imputation for the missing values management, whereas the others did not plan any.

## Discussion

In this systematic review, sample size estimation for assessment of VFDs in critical care trials was heterogeneous and not in adequacy with the actual distribution of VFDs. There was also important heterogeneity in the definitions of VFDs and in the methods used for sample size estimation among trials. Sample size estimation extends beyond the VFDs to all medical fields. Indeed, it is essential to have the right estimate before beginning a trial because of several aspects, such as ethical, logistic, and financial concerns. When there is heterogeneity of both outcome definition and methods for calculating it, the sample size may be underestimated and lack the power to show a clinically meaningful difference, or it may be overestimated and waste resources and expose an unnecessary number of subjects to a potentially harmful treatment, or deny a potentially beneficial one [11–13].

### Sample size estimation: consensus definition of the outcome

Following Contentin et al. [16] and Yehya et al. [8], we found important differences between definitions of VFDs across trials, thus making it difficult to conduct meta-analyses, as there is no common core.

Yehya et al. made several recommendations, including on how to explicitly define VFDs [8]. Hence, a core outcome set, such as the Core Outcomes in Ventilation Trials [51], could be used. This includes standardized definitions and measures for extubation, reintubation, duration of mechanical ventilation, and mortality. However, although Blackwood et al. defined several components of VFDs, there was no consensual definition of VFDs. In future studies, all components of this outcome should be reported to facilitate the preparation of the future statistical analysis plan, especially for the sample size estimation because it includes some of these components.

Finally, other alternative approaches should be considered such as a ranked composite score used in The Esophageal Pressure-Guided Ventilation 2 trial (EPVent-2) [52]. Alive and ventilator-free (AVF), the primary outcome used in EPVent-2, is a recent hierarchical composite outcome that does not treat mortality as equivalent to prolonged intubation [7]. This kind of outcome is already applied to other disciplines than critical care, such as in lung and cardiovascular clinical trials [53, 54]. In a simulation-based study [7], AVF had higher power to detect differences in mortality than VFDs. Consequently, the sample size could be lower with this outcome when there is a difference in mortality. Moreover, this outcome typically requires fewer patients because its distribution is closer to a Gaussian distribution than the distribution of VFDs. Finally, unlike AVF, which takes clinical priorities into account, VFDs treat death or remaining intubated in the same way (i.e., if they were of equal relevance). In contrast, death is considered more important than the duration of mechanical ventilation in AVF, which seems more clinically relevant.

### Sample size estimation: methods

Several parameters are required to estimate the sample size of an RCT. First is the tail of the risk (one- or two-sided). In most superiority studies from our review, the authors used a two-tailed test. However, if a specific and unidirectional difference is hypothesized (e.g., treatment *vs* placebo), a one-sided risk should be preferred to test the null hypothesis for the two groups [55].

We also need the expected difference (i.e., the effect size) between two groups in a superiority study or a loss of efficacy in a noninferiority study. Some studies from our review used a superiority margin smaller than the noninferiority margin, which is hardly justifiable. Furthermore, VFDs do not follow a Gaussian distribution and using the median difference as the effect size when estimating sample size seems more relevant than using the mean difference. In addition, the expected standard deviation was heterogeneous across studies. Even if the population was different, the standard deviation should not have such a large difference for the same outcome. A consensus according to the context to choose the correct effect size and the related standard deviation seems to be necessary to estimate the right sample size and ensure sufficient powered.

Finally, 73% of the included studies had chosen a power of 80% for sample size estimation. Therefore, if the other parameters for this estimation are under- or overestimated, there is limited room for mistake.

### Sample size estimation: statistical methods for ventilator-free days

There are two concepts to consider when using VFDs: their distribution and the presence of competitive risks. However, there are rarely reported, which may contribute to the fact that most included trials were underpowered.

First, the unique distribution of VFDs could make their statistical analysis more problematic. Indeed, some articles reported a zero-inflated beta distribution [30, 31]. For this distribution, a GLMM [31, 43] (e.g., with a zero-inflated beta model or hurdle-negative binomial model [56]) or a GAMLSS [30] can be used. These models also allow for adjusting covariates, thus reducing the sample size and increasing the power [57]. Here, VFDs are treated as a count outcome, where death, intubation, and extubation are treated together and combined as one entity. These models assess whether there is a difference between groups on distribution. However, there was an important heterogeneity in the reported distribution of VFDs and in the tests used among trials, which prompts further clarification.

Second, regarding the presence of competitive risks, Yehya et al. [8] recommended reporting the mortality because the number of VFDs combines mortality and the duration of ventilation. Because mortality is a competitive event of extubation, competing risk regression using the Fine and Gray regression or the Cox-specific regression seems more appropriate than the usual tests, such as the Student *t*-test or Mann–Whitney–Wilcoxon rank-sum test [15]. In addition, not taking mortality into account may underpower the study, especially if mortality is low. Moreover, these tests enable adjustment for covariates and interim monitoring, which are common in RCTs. Nevertheless, the cumulative incidence function provided by the Fine and Gray regression does not have a natural interpretation [58] and does not take the zero inflation into account. Here, VFDs are treated as a time-to-event outcome. However, in our opinion, this does not express the real definition of VFDs. Indeed, it is more the extubation time that is shaped, with death as a competitive risk.

As a result, these two types of models are based on two distinct concepts: a count outcome or a time-to-event outcome, not comparing the same things. Indeed, as mentioned above, the different parts of the VFDs components (i.e., death and ventilation duration) have a different importance depending on the type of model used, which is not much discussed in the literature. However, a recent study looked into the count outcome [59]. No model was globally recommended, and the best model depends above all on the expected data distribution. However, to date, there is no clear answer as to which statistical test to use.

Nevertheless, based on the current results, we believe that the usual tests should be discouraged for analysis of VFDs and more complex models considering competitive risks, the unique distribution of VFDs, and any covariates, such as centers, might be more appropriate for better fit the data.

### Sample size estimation: recent methodological approaches

Common formulae can be used to estimate a trial’s sample size [13]. However, for complex models such as the GLMM and GAMLSS, these formulae cannot be employed, and simulation-based power analyses could be useful [60].

The Markov chain model is another interesting approach to stochastically describe a sequence of possible events in which the probability of each event depends only on the state attained by the previous event [61]. In the case of VFDs, three possible states could be defined: intubated, extubated, or dead.

The use of simulations to estimate the sample size should probably be encouraged, especially for VFDs, given their complex probability distribution [8].

### Limitations

We only selected studies involving adults to focus on one type of population and reduce population heterogeneity. Therefore, only RCTs were included because these studies must report sample size estimation and are less prone to bias. Moreover, because we knew there was heterogeneity in how VFDs are defined, we selected studies published only in journals with a 2021 impact factor greater than five in the hopes of a more rigorous methodology. However, in this systematic review, we found few unbiased studies.

The selected studies included different populations, mainly because the inclusion criteria of the reported studies were quite different, thus reducing the possibility of generalizing our results. However, we focused on sample size estimation, which was not affected by differences between studies. Moreover, some data of interest were not reported in many studies, which restrained sample size estimation. Additionally, Harhay et al. [6] found that 63% of the power parameters were unreported in selected RCTs.

### Strengths

To our knowledge, this is the first systematic review of 26 RCTs assessing how sample sizes are estimated in trials with VFDs as the primary outcome. First, we followed the PRISMA 2020 statement guidelines with checklists [17] (Additional file 1: Tables S8 and S9). Second, two investigators independently reviewed the studies. Third, we used two databases to be as comprehensive as possible. In addition, we did not place any limit on the publication year: All studies referenced since the creation of the database were therefore included but restricted to journals with an impact factor greater than five. Finally, we used the RoB2 tool to evaluate any potential biases.

## Conclusions

In this systematic review of RCTs with VFDs as the primary outcome, we observed strong variability in the methods and results of sample size estimation, in addition to heterogeneity in the definitions of VFDs. Moreover, the uncommon distribution of the number of VFDs in clinical trials may have important implications which warrants further investigation, such as for sample size estimation and analysis. Complex models and simulation might be useful for sample size estimation when using VFDs as a primary outcome in future trials. The methods used are of great importance as they directly impact the number of patients to enroll and could jeopardize the feasibility of a trial, due to ethical, logistical, and financial reasons.

## Supplementary Information

**Additional file 1.** Additional file 1 of Sample size estimation in clinical trials using ventilator-free days as the primary outcome: a systematic review. **Appendix 1.** Searching terms used for reviewing. **Appendix 2.** Extracted data. **Appendix 3.** Statistical analysis plan. **Appendix 4.** Packages of R. **Table S1.** Characteristics of the included studies (for each study). **Table S2.** Risk-of-bias summary: review authors’ judgments about each risk-of-bias item based on Revised Cochrane risk-of-bias tools for randomized trials (RoB2). **Table S3.** Parameters reported for the sample size estimation of ventilator-free days (for each study). **Table S4.** Sample size estimation according to statistical test analysis, based on expected parameters and effect size. **Table S5.** Observed ventilator-free days and mortality rate according to group. **Table S6.** Sample size estimation according to statistical test analysis, based on observed parameters, and author’s conclusion. **Table S7.** Sample size estimation from the simulation of Bodet-Contentin et al. according to the statistical test analysis. **Table S8.** PRISMA 2020 Article Checklist. **Table S9.** PRISMA 2020 Abstract Checklist. **Figure S1.** PRISMA flow diagram from search in December 2021. **Figure S2.** Expected mean difference in ventilator-free days and related standard deviation in the 26 studies included in the systematic review.

## Data Availability

The datasets used and/or analyzed during the current study are available from the corresponding author on reasonable request. An annotated R code for sample size simulations is available in open access in Zenodo [62].

## Abbreviations

EPVent-2: The Esophageal Pressure-Guided Ventilation 2 trial
GAMLSS: Generalized additive model for location, scale, and shape
GLMM: Generalized linear mixed model
IQR: Interquartile range
PRISMA: Preferred Reporting Items for Systematic Reviews and Meta-Analyses
RCTs: Randomized clinical trials
RoB2: Version 2 of the Cochrane risk-of-bias tool
VFDs: Ventilator-free days
ZINB: Zero-inflated negative binomial
