# Supplementary material for: Sample size estimation in clinical trials using ventilator-free days as the primary outcome: a systematic review
Source: Crit Care. 2023 Aug 1;27:303. doi: 10.1186/s13054-023-04562-y (PMC10394791; doi:10.1186/s13054-023-04562-y)
Supplement: Supplementary file 1 — Additional file 1. Additional file 1 of Sample size estimation in clinical trials using ventilator-free days as the primary outcome: a systematic review. Appendix 1. Searching terms used for reviewing. Appendix 2. Extracted data. Appendix 3. Statistical analysis plan. Appendix 4. Packages of R. Table S1. Characteristics of the included studies (for each study). Table S2. Risk-of-bias summary: review authors’ judgments about each risk-of-bias item based on Revised Cochrane risk-of-bias tools for randomized trials (RoB2). Table S3. Parameters reported for the sample size estimation of ventilator-free days (for each study). Table S4. Sample size estimation according to statistical test analysis, based on expected parameters and effect size. Table S5. Observed ventilator-free days and mortality rate according to group. Table S6. Sample size estimation according to statistical test analysis, based on observed parameters, and author’s conclusion. Table S7. Sample size estimation from the simulation of Bodet-Contentin et al. according to the statistical test analysis. Table S8. PRISMA 2020 Article Checklist. Table S9. PRISMA 2020 Abstract Checklist. Figure S1. PRISMA flow diagram from search in December 2021. Figure S2. Expected mean difference in ventilator-free days and related standard deviation in the 26 studies included in the systematic review. [file 13054_2023_4562_MOESM1_ESM.docx]

Additional file 1

Sample size estimation in clinical trials using Ventilator-free days as the primary outcome: A systematic review.

Laurent Renard Triché^1^, Emmanuel Futier^1,2^, Manuela De Carvalho^3^, Nathalie Piñol-Domenech^3^, Laëtitia Bodet-Contentin^4,5^, Matthieu Jabaudon^1,2^, Bruno Pereira^6^.

^1^Department of Perioperative Medicine, CHU Clermont-Ferrand, Clermont-Ferrand, France

^2^iGReD, CNRS, INSERM, Université Clermont Auvergne, Clermont-Ferrand, France

^3^Université Clermont Auvergne, Health Library, Clermont-Ferrand, France

^4^Medical Intensive Care Unit, CHRU de Tours, France

^5^INSERM, SPHERE, UMR1246, Université de Tours et Nantes, Tours et Nantes, France

^6^Biostatistics Unit, Department of Clinical Research, and Innovation (DRCI), CHU Clermont-Ferrand, Clermont-Ferrand, France

Corresponding Author:

Laurent Renard Triché
Department of Perioperative Medicine, CHU Clermont-Ferrand
58 rue Montalembert, 63 000 Clermont-Ferrand, France
+33 6 67 05 08 74
lrenard--triche@chu-clermontferrand.fr

Table of contents

Methods

Appendix 1. Searching terms used for reviewing.

Appendix 2. Extracted data.

Appendix 3. Statistical analysis plan.

Appendix 4. Packages of R.

References

Tables

Table S1. Characteristics of the included studies (for each study).

Table S2. Risk-of-bias summary: review authors’ judgments about each risk-of-bias item based on Revised Cochrane risk-of-bias tools for randomized trials (RoB2).

Table S3. Parameters reported for the sample size estimation of ventilator-free days (for each study).

Table S4. Sample size estimation according to statistical test analysis, based on expected parameters and effect size.

Table S5. Observed ventilator-free days and mortality rate according to group.

Table S6. Sample size estimation according to statistical test analysis, based on observed parameters, and author’s conclusion.

Table S7. Sample size estimation from the simulation of Bodet-Contentin et al. according to the statistical test analysis.

Table S8. PRISMA 2020 Article Checklist.

Table S9. PRISMA 2020 Abstract Checklist.

Figures

Figure S1. PRISMA flow diagram from search in December 2021.

Figure S2. Expected mean difference in ventilator-free days and related standard deviation in the 26 studies included in the systematic review.

Methods

Appendix 1. Searching terms used for reviewing.

The search strategies were developed with the help of two university librarians (MdC and NPD). We searched through two databases: MEDLINE via PubMed and Embase. The search strategies used a combination of keywords related to VFDs, the names of journals with a 2021 impact factor greater than five, and study type, from inception to December 2021. Two investigators (LRT and MJ) independently screened the titles and abstracts of the search results.

MEDLINE via PubMed

("Ventilator free days"[TW] OR "Ventilator free day"[TW] OR "Ventilation free days"[TW] OR "MV-free days"[TW] OR (Ventilat*[Title] AND free[Title] AND (days[Title] OR day[Title])))

AND

("the new england journal of medicine"[Journal] OR "Lancet (London, England)"[Journal] OR "Nature"[Journal] OR "Nature medicine"[Journal] OR "Science (New York, N.Y.)"[Journal] OR "JAMA"[Journal] OR "Annals of internal medicine"[Journal] OR "plos medicine"[Journal] OR "BMJ (Clinical research ed.)"[Journal] OR "BMJ"[journal] OR "american journal of respiratory and critical care medicine"[Journal] OR "Chest"[Journal] OR "Critical care (London, England)"[Journal] OR "Critical care medicine"[Journal] OR "Intensive care medicine"[Journal] OR "The Lancet. Respiratory medicine"[Journal] OR "Nature immunology"[Journal] OR "Nature reviews. Immunology"[Journal])

AND

(randomized controlled trial[pt] OR controlled clinical trial[pt] OR randomized[TW] OR placebo[TW] OR clinical trials as topic[mesh:noexp] OR randomly[TW] OR trial[TW])

Embase

(('ventilator free days' OR 'ventilator-free days' OR 'ventilator free day' OR 'ventilation free days' OR 'mv-free days' OR (ventilat*:ti AND free:ti AND (days:ti OR day:ti))) AND ('new england journal of medicine':jt OR 'nature'/jt OR 'lancet (london, england)'/jt OR 'the lancet'/jt OR 'nature medicine':jt OR 'science'/jt OR 'science (new york, n.y.)'/jt OR 'jama'/jt OR 'jama - journal of the american medical association'/jt OR 'jama : the journal of the american medical association'/jt OR 'annals of internal medicine':jt OR 'plos medicine'/jt OR 'bmj'/jt OR 'bmj (clinical research ed.)'/jt OR 'bmj (online)'/jt OR 'the bmj'/jt OR 'american journal of respiratory and critical care medicine'/jt OR 'chest'/jt OR 'critical care'/jt OR 'critical care (london, england)'/jt OR 'critical care medicine'/jt OR 'intensive care medicine'/jt OR 'the lancet respiratory medicine'/jt OR 'nature immunology':jt OR 'nature reviews immunology'/jt)) AND ('article'/it OR 'article in press'/it)

Appendix 2. Extracted data.

Features

PMID (PubMed IDentifier) if available, year of publication, design, definition of ventilator-free days (VFDs), type of ventilation, onset of VFDs, length of VFDs, PaO_2_/FiO_2_ ratio.

Methods

Power, $\alpha$ risk, inflation $\alpha$ risk, type of study (superiority/noninferiority/equivalence), type of test (unilateral/bilateral), type of analysis (intention-to-treat/per-protocol), margin of noninferiority if applicable, VFDs expected in control and experimental groups, VFDs’ distribution and correction if applicable, mortality rate expected in control and experimental groups, expected difference between groups and justification, expected dropout patients, management of missing values, statistical method for sample size estimation and for analysis of the primary outcome of VFDs, sample size estimation.

Results

Sample size, missing values, premature cessation, dropout patients, VFDs in control and experimental groups, mortality rate in control and experimental groups, difference between groups and confidence interval, effect size, *p-value*.

Research Electronic Data Capture (REDCap) hosted at CHU Clermont-Ferrand[1,2]

REDCap is a secure, web-based software platform designed to support data capture for research studies, providing 1) an intuitive interface for validated data capture, 2) audit trails for tracking data manipulation and export procedures, 3) automated export procedures for seamless data downloads to common statistical packages, and 4) procedures for data integration and interoperability with external sources.

Appendix 3. Statistical analysis plan.

All sample size estimation were achieved with R version 4.2.0[3] using the following equations with $\alpha$: type I error, $\left( 1-\beta\right)$: statistical power, $n$: size of one group and $N$: size of all groups.

For these estimations, we used the DaCCoTA Project[4] and the Sample Size Calculations in Clinical Research[5].

When the mean is missing, we estimated it from the median, the interquartile range, and the sample size[6].

First, we calculated the sample size with the expected parameters displayed in the Material and Methods section (corresponding to the estimated sample size), with the different statistical tests presented below. Second, we calculated the sample size with the observed parameters displayed in the Results section (corresponding to the observed sample size), with the same statistical tests. We estimated these two sample sizes because we wanted to highlight the estimated sample size and the observed sample size. Furthermore, some values needed to estimate sample size were not available in the Materials and Methods sections (e.g., VFDs in the control group and their standard deviation). Hence, with the observed parameters, we hoped to estimate more sample sizes, to show the range of sample size according to the different tests.

The different statistical tests were as follows: the Student *t*-test and the Mann–Whitney–Wilcoxon rank-sum test because these are standard tests used in several studies; the Mann–Whitney–Wilcoxon rank-sum test using the Noether formula to compare if the result differ from the previous one; the Cox regression because the VFDs are a time-dependent event; the zero-inflated negative binomial regression because the VFDs involve a zero-inflation; and finally, the Fine and Gray regression because the VFDs involve a competitive risk.

Student *t*-test based on Gaussian distribution[5]

For the sample size estimation based on expected parameters, we used the standard deviation of the control group when it was available, displayed in the Material and Methods section.

For the sample size estimation based on observational parameters, we used the pooled standard deviation $\sigma$:

$\sqrt{\left( \frac{{\sigma_{1}}^{2}+{\sigma_{2}}^{2}}{2} \right)}$, where $\sigma_{1}$ is the standard deviation of the control group and $\sigma_{2}$ the standard deviation of the interventional group, given that the two $\sigma$ are available in the Results section.

For the sample size estimation, we used the following equation:

$n= \frac{2 \sigma^{2}}{\Delta^{2}}\left( Z_{1- \frac{\alpha}{2}}+Z_{1- \beta} \right)^{2}$, with $\sigma$ the standard deviation and $\Delta$ the absolute difference between groups.

We used the pwr package to estimate the sample size for the Student *t*-test for the superiority studies.

For the noninferiority study, we used the following equation according to one group:

$n= \frac{2 \sigma^{2}}{\left( \Delta- \delta\right)^{2}}\left( Z_{1- \frac{\alpha}{2}}+Z_{1- \beta} \right)^{2}$, with $\delta$ the margin of the noninferiority.

We used the TrialSize package to estimate the sample size for the Student *t*-test for the noninferiority studies.

Mann–Whitney–Wilcoxon[7]

This nonparametric test uses the sample size estimation of the Student *t*-test. We therefore simply multiplied the sample size estimated according to the Student *t*-test by 1.15 to have an increase of 15% in the sample size.

Noether formula[8,9]

First, we estimated the effect size $\frac{\delta}{\sigma}= \frac{\mu_{1}- \mu_{2}}{\sigma}$ , where $\delta$ is the absolute difference between groups, $\sigma$ the standard deviation, $\mu_{1}$ the mean of the control group and $\mu_{2}$ the standard deviation of the interventional group.

Then, we calculated the relative effect: there is a relation between the corresponding relative effect $p$ and the effect size for a normal distribution.


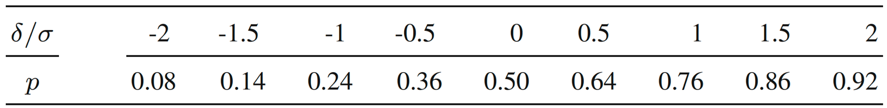
Finally, for the sample size estimation, we used the following equation:

$N= \frac{\left( Z_{\alpha}+Z_{\beta} \right)^{2}}{12c(1-c){(p - \frac{1}{2})}^{2}}$ , where c is the proportion of subjects in the control group and p the relative effect.

We used the rankFD package to estimate the sample size for the Mann–Whitney–Wilcoxon rank-sum test using the Noether formula.

One-way ANOVA (ANalysis Of VAriance)[10]

We used ANOVA when there were 3 or more groups in the selected studies.

We estimated first the Cohen’s $f$ effect size:

$f= \frac{\sigma_{m}}{\sigma}$ , where $\sigma_{m}=\sqrt{\frac{\sum_{i=1}^{k} \left( \left( m_{i} - m \right)^{2} \right)}{k}}$ , with $k$ the number of groups, $m_{i}$ the mean of each group and $m$ the grand mean corresponding to the mean of the means, and $\sigma$ the pooled standard deviation.

Then, for the sample size estimation, we used the following equation:

$n= \frac{n_{.05}}{400 \times{(f}^{2})}+1$ , where $n_{.05}$ is the necessary sample size for the given $\alpha$, $u$ (the degree of freedom i.e., $k-1$) and desired power $\beta$ at $f=0.5$ read from the Table.

We used the pwr package to estimate the sample size for the one-way ANOVA.

Kruskal-Wallis[7]

We used Kruskal-Wallis when there were 3 or more groups in the selected studies. This nonparametric test uses the sample size estimation of the ANOVA. Like above, we multiply the sample size estimated according to the ANOVA test by 1.15 to have an increase of 15% in the sample size.

Cox regression[5,11]

We did not use the log-rank test because testing superiority or noninferiority is difficult to be carried out[5].

For the sample size estimation, we used the following equation:

$N= \frac{\left( Z_{1- \frac{\alpha}{2}}+Z_{1- \beta} \right)^{2}}{{(log \left( HR \right)- \delta)}^{2}\times p_{e}\times p_{c} \times d}$ , where $HR$ is the Hazard Ratio of the VFDs, $\delta$ the margin of the noninferiority if applicable, $p_{e}$ the proportion of patients within the experimental group, $p_{c}$ the proportion of patients within the control group and $d$ the probability of observing an event.

We used the TrialSize package to estimate the sample size for the Cox regression.

When the HR isn’t available, we estimated it as follows:

$\frac{\mu({VFD}_{e})}{\mu({VFD}_{c})}$ , where ${\mu(VFD}_{e})$ is the mean of the VFDs in the experimental group and ${\mu(VFD}_{c})$ the mean of the VFDs in the control group, as described by Zhu and Lakkis.

Zero-inflated negative binomial regression[11]

First, we calculated the mean rates of events ratio:

$r= \frac{\mu({VFD}_{E})}{\mu({VFD}_{C})}$ , where ${\mu(VFD}_{E})$ is the mean of the VFDs in the experimental group and ${\mu(VFD}_{C})$ the mean of the VFDs in the control group.

Then, we estimated the overdispersion $\kappa$ by using the simulation of Bodet-Contentin et al.[12] and the meta-analysis of Jabaudon et al.[13] with a generalized linear mixed model. The parameters of this model were the negative binomial family with a link logit and a zero inflation. The center was considered as random effect. We found the same overdispersion in the two studies: $\kappa=0.057$.

For the sample size estimation, we used the “approach 2” as recommended in their article:

$n= \frac{\left( Z_{\frac{\alpha}{2}} \sqrt{V_{0|2}}+ Z_{\beta}\sqrt{V_{A}} \right)^{2}}{\left( \log r \right)^{2}}$ ,

$V_{A}$ is the variance estimated under the alternative hypothesis:

$V_{A}= \frac{1}{\mu_{t}}\left( \frac{1}{\lambda_{C}}+ \frac{1}{\lambda_{E}\theta} \right)+ \frac{(1+\theta)\kappa}{\theta}$ , where $\mu_{t}$ is the average subject exposure time (same unit as the one used for the mean event rate per time ($\lambda$)), $\lambda_{C}$ the mean event rate per time in the control group, $\lambda_{E}$ the mean event rate per time in the interventional group and $\Theta=\frac{n_{E}}{n_{C}}$ the allocation ratio with $n_{E}$ the sample size in the interventional group and $n_{C}$ the sample size in the control group.

$V_{0|2}$ is the variance estimated under the null hypothesis according to the method 2 using the true event rates ( ${\lambda_{C}= \mu(VFD}_{C})$ and ${\lambda_{E}= \mu(VFD}_{E})$ ):

$$V_{O|2}=V_{A}$$

We used the MKmisc package to estimate the sample size for the zero-inflated negative binomial regression.

Fine and Gray regression[14]

First, we estimated the $\Xi$ corresponding of:

$\Xi= \frac{\lambda_{1E}\left( t \right) + \lambda_{2E}(t)}{\lambda_{1C}\left( t \right) + \lambda_{2C}(t)}$ , where $\lambda_{1E}$ is the proportion of patients within the experimental group with the event of interest (i.e. extubation), $\lambda_{2E}$ is the proportion of patients within the experimental group with the competing event (i.e. death), $\lambda_{1C}$ is the proportion of patients within the control group with the event of interest and $\lambda_{2C}$ is the proportion of patients within the control group with the competing event.

Then, for the sample size estimation, we used the following equation:

$N= \frac{\left( Z_{1- \frac{\alpha}{2}}+Z_{1- \beta} \right)^{2}}{{(log \left( \Xi\right)- \delta)}^{2}\times p_{E}\times p_{C} \times d}$ , where $\delta$ is the margin of the noninferiority if applicable, $p_{E}$ the proportion of patients within the experimental group, $p_{C}$ the proportion of patients within the control group and $d$ the probability of observing an event.

Because the package to estimate the sample size for a Fine and Gray regression is not available in R, we built it and it will be made available upon reasonable request.

Appendix 4. Packages of R[3].

Hmisc for label and describe.

Harrell FEJ. Hmisc: Harrell Miscellaneous. 2022 R package version 4.7-0. Available from: https://CRAN.R-project.org/package=Hmisc.

pwr for the sample size estimation of the superiority study using Student *t*-test.

Champely S. pwr: Basic Functions for Power Analysis. 2020 R package version 1.3-0. Available from: https://CRAN.R-project.org/package=pwr.

TrialSize for the sample size estimation of the noninferiority studies using Student *t*-test or the Cox regression.

Zhang E, Wu VQ, Chow SC, Zhang HG. TrialSize: R Functions for Chapter 3, 4, 6, 7, 9, 10, 11, 12, 14, 15 of Sample Size Calculation in Clinical Research. 2020 R package version 1.4. Available from: https://CRAN.R-project.org/package=TrialSize.

rankFD for the sample size estimation using Noether formula.

Konietschke F, Friedrich S, Brunner E, Pauly M. rankFD: Rank-Based Tests for General Factorial Designs. 2022 R package version 0.1.1. Available from: https://CRAN.R‑project.org/package=rankFD.

MKmisc for the sample size estimation using the zero-inflated negative binomial regression.

Kohl M. MKmisc: Miscellaneous functions from M. Kohl. 2021 R package version 1.8. Available from: https://www.stamats.de.

readxl to read Excel files.

Wickham H, Bryan J. readxl: Read Excel Files. 2022 R package version 1.4.0. Available from: https://CRAN.R-project.org/package=readxl.

glmmTMB to use a GLMM (Generalized Linear Mixed Model).

Brooks ME, Kristensen K, van Benthem KJ, Magnusson A, Berg CW, Nielsen A, et al. glmmTMB Balances Speed and Flexibility Among Packages for Zero-inflated Generalized Linear Mixed Modeling. The R Journal. 2017 Dec;9(2):378-400.

PRISMA2020 for the DiagrammeR.

Haddaway NR, Page MJ, Pritchard CC, McGuinness LA. PRISMA2020: An R package and Shiny app for producing PRISMA 2020-compliant flow diagrams, with interactivity for optimised digital transparency and Open Synthesis. Campbell Syst Rev. 2022 Jun;18(2):e1230.

References

1. Harris PA, Taylor R, Thielke R, Payne J, Gonzalez N, Conde JG. Research electronic data capture (REDCap) -- a metadata-driven methodology and workflow process for providing translational research informatics support. J Biomed Inform. 2009;42(2):377–81. doi:10.1016/j.jbi.2008.08.010

2. Harris PA, Taylor R, Minor BL, Elliott V, Fernandez M, O’Neal L, et al. The REDCap consortium: Building an international community of software platform partners. J Biomed Inform. 2019;95:103208. doi:10.1016/j.jbi.2019.103208

3. R Core Team. R: A Language and Environment for Statistical Computing [Internet]. Vienna Austria R Found. Stat. Comput. https://www.r-project.org. Accessed 13 Feb 2022.

4. DACCOTA Statistical Resources. https://med.und.edu/daccota/berdc-resources.html. Accessed 6 Nov 2022.

5. Chow S-C, Shao J, Wang H, Lokhnygina Y. Sample Size Calculations in Clinical Research. 3rd ed. New York: Chapman and Hall/CRC; 2017. doi:10.1201/9781315183084

6. Wan X, Wang W, Liu J, Tong T. Estimating the sample mean and standard deviation from the sample size, median, range and/or interquartile range. BMC Med Res Methodol. 2014;14:135. doi:10.1186/1471-2288-14-135

7. Lehmann EL. Nonparametrics: statistical methods based on ranks. San Francisco: Holden-Day; 1975.

8. Noether GE. Sample Size Determination for Some Common Nonparametric Tests. J Am Stat Assoc. 1987;82(398):645–7. doi:10.2307/2289477

9. Brunner E, Bathke AC, Konietschke F. Rank and pseudo-rank procedures for independent observations in factorial designs: using R and SAS. Springer Cham; 2018. https://doi.org/10.1007/978-3-030-02914-2

10. Cohen J. Statistical Power Analysis for the Behavioral Sciences. 2nd ed. New York: Routledge; 1988. doi:10.4324/9780203771587

11. Zhu H, Lakkis H. Sample size calculation for comparing two negative binomial rates. Stat Med. 2014;33(3):376–87. doi:10.1002/sim.5947

12. Bodet-Contentin L, Frasca D, Tavernier E, Feuillet F, Foucher Y, Giraudeau B. Ventilator-Free Day Outcomes Can Be Misleading: Crit Care Med. 2018;46(3):425–9. doi:10.1097/CCM.0000000000002890

13. Jabaudon M, Blondonnet R, Pereira B, Cartin-Ceba R, Lichtenstern C, Mauri T, et al. Plasma sRAGE is independently associated with increased mortality in ARDS: a meta-analysis of individual patient data. Intensive Care Med. 2018;44(9):1388–99. doi:10.1007/s00134-018-5327-1

14. Schulgen G, Olschewski M, Krane V, Wanner C, Ruf G, Schumacher M. Sample sizes for clinical trials with time-to-event endpoints and competing risks. Contemp Clin Trials. 2005;26(3):386–96. doi:10.1016/j.cct.2005.01.010

15. Morris PE, Papadakos P, Russell JA, Wunderink R, Schuster DP, Truwit JD, et al. A double-blind placebo-controlled study to evaluate the safety and efficacy of L-2-oxothiazolidine-4-carboxylic acid in the treatment of patients with acute respiratory distress syndrome. Crit Care Med. 2008;36(3):782–8. doi:10.1097/CCM.0B013E318164E7E4

16. Paine R, Standiford TJ, Dechert RE, Moss M, Martin GS, Rosenberg AL, et al. A randomized trial of recombinant human GM-CSF for patients with acute lung injury. Crit Care Med. 2012;40(1):90–7. doi:10.1097/CCM.0b013e31822d7bf0

17. Mackle D, Bellomo R, Bailey M, Beasley R, Deane A, Eastwood G, et al. Conservative Oxygen Therapy during Mechanical Ventilation in the ICU. N Engl J Med. Massachusetts Medical Society; 2020;382(11):989–98. doi:10.1056/NEJMoa1903297

18. Villar J, Ferrando C, Martínez D, Ambrós A, Muñoz T, Soler JA, et al. Dexamethasone treatment for the acute respiratory distress syndrome: a multicentre, randomised controlled trial. Lancet Respir Med. 2020;8(3):267–76. doi:10.1016/S2213-2600(19)30417-5

19. Zhou Y, Jin X, Lv Y, Wang P, Yang Y, Liang G, et al. Early application of airway pressure release ventilation may reduce the duration of mechanical ventilation in acute respiratory distress syndrome. Intensive Care Med. 2017;43(11):1648–59. doi:10.1007/s00134-017-4912-z

20. Trouillet J-L, Luyt C-E, Guiguet M, Ouattara A, Vaissier E, Makri R, et al. Early Percutaneous Tracheotomy Versus Prolonged Intubation of Mechanically Ventilated Patients After Cardiac Surgery. Ann Intern Med. 2011;154(6):373–83. doi:10.7326/0003-4819-154-6-201103150-00002

21. Simonis FD, Neto AS, Binnekade JM, Braber A, Bruin KCM, Determann RM, et al. Effect of a Low vs Intermediate Tidal Volume Strategy on Ventilator-Free Days in Intensive Care Unit Patients Without ARDS: A Randomized Clinical Trial. JAMA. 2018;320(18):1872–80. doi:10.1001/jama.2018.14280

22. Algera AG, Pisani L, Neto AS, Gama de Abreu M, Pelosi P, Schultz MJ, et al. Effect of a Lower vs Higher Positive End-Expiratory Pressure Strategy on Ventilator-Free Days in ICU Patients Without ARDS: A Randomized Clinical Trial. JAMA. 2020;324(24):2509–20. doi:10.1001/jama.2020.23517

23. Tomazini BM, Maia IS, Cavalcanti AB, Berwanger O, Rosa RG, Veiga VC, et al. Effect of Dexamethasone on Days Alive and Ventilator-Free in Patients With Moderate or Severe Acute Respiratory Distress Syndrome and COVID-19: The CoDEX Randomized Clinical Trial. JAMA. 2020;324(13):1307–16. doi:10.1001/jama.2020.17021

24. Grieco DL, Menga LS, Cesarano M, Rosà T, Spadaro S, Bitondo MM, et al. Effect of Helmet Noninvasive Ventilation vs High-Flow Nasal Oxygen on Days Free of Respiratory Support in Patients With COVID-19 and Moderate to Severe Hypoxemic Respiratory Failure: The HENIVOT Randomized Clinical Trial. JAMA. 2021;325(17):1731–43. doi:10.1001/jama.2021.4682

25. van Meenen DMP, van der Hoeven SM, Binnekade JM, de Borgie CAJM, Merkus MP, Bosch FH, et al. Effect of On-Demand vs Routine Nebulization of Acetylcysteine With Salbutamol on Ventilator-Free Days in Intensive Care Unit Patients Receiving Invasive Ventilation: A Randomized Clinical Trial. JAMA. 2018;319(10):993–1001. doi:10.1001/jama.2018.0949

26. Spragg RG, Lewis JF, Walmrath H-D, Johannigman J, Bellingan G, Laterre P-F, et al. Effect of Recombinant Surfactant Protein C–Based Surfactant on the Acute Respiratory Distress Syndrome. N Engl J Med. 2004;351(9):884–92. doi:10.1056/NEJMoa033181

27. Welte T, Dellinger RP, Ebelt H, Ferrer M, Opal SM, Singer M, et al. Efficacy and safety of trimodulin, a novel polyclonal antibody preparation, in patients with severe community-acquired pneumonia: a randomized, placebo-controlled, double-blind, multicenter, phase II trial (CIGMA study). Intensive Care Med. 2018;44(4):438–48. doi:10.1007/s00134-018-5143-7

28. Rice TW, Wheeler AP, Thompson BT, deBoisblanc BP, Steingrub J, Rock P, et al. Enteral Omega-3 Fatty Acid, γ-Linolenic Acid, and Antioxidant Supplementation in Acute Lung Injury. JAMA. 2011;306(14):1574–81. doi:10.1001/jama.2011.1435

29. Bernard GR, Francois B, Mira J-P, Vincent J-L, Dellinger RP, Russell JA, et al. Evaluating the Efficacy and Safety of Two Doses of the Polyclonal Anti-Tumor Necrosis Factor-α Fragment Antibody AZD9773 in Adult Patients With Severe Sepsis and/or Septic Shock: Randomized, Double-Blind, Placebo-Controlled Phase IIb Study*. Crit Care Med. 2014;42(3):504–11. doi:10.1097/CCM.0000000000000043

30. Chung KK, Wolf SE, Renz EM, Allan PF, Aden JK, Merrill GA, et al. High-frequency percussive ventilation and low tidal volume ventilation in burns: A randomized controlled trial. Crit Care Med. 2010;38(10):1970–7. doi:10.1097/CCM.0b013e3181eb9d0b

31. Rice TW, Wheeler AP, Thompson BT, Steingrub J, Hite RD, Moss M, et al. Initial Trophic vs Full Enteral Feeding in Patients With Acute Lung Injury: The EDEN Randomized Trial. JAMA. 2012;307(8):795–803. doi:10.1001/jama.2012.137

32. Bein T, Weber-Carstens S, Goldmann A, Müller T, Staudinger T, Brederlau J, et al. Lower tidal volume strategy (≈3 ml/kg) combined with extracorporeal CO2 removal versus ‘conventional’ protective ventilation (6 ml/kg) in severe ARDS: the prospective randomized Xtravent-study. Intensive Care Med. 2013;39(5):847–56. doi:10.1007/s00134-012-2787-6

33. Hodgson CL, Cooper DJ, Arabi Y, King V, Bersten A, Bihari S, et al. Maximal Recruitment Open Lung Ventilation in Acute Respiratory Distress Syndrome (PHARLAP). A Phase II, Multicenter Randomized Controlled Clinical Trial. Am J Respir Crit Care Med. 2019;200(11):1363–72. doi:10.1164/rccm.201901-0109OC

34. Kacmarek RM, Villar J, Parrilla D, Alba F, Solano R, Liu S, et al. Neurally adjusted ventilatory assist in acute respiratory failure: a randomized controlled trial. Intensive Care Med. 2020;46(12):2327–37. doi:10.1007/s00134-020-06181-5

35. Kacmarek RM, Wiedemann HP, Lavin PT, Wedel MK, Tütüncü AS, Slutsky AS. Partial Liquid Ventilation in Adult Patients with Acute Respiratory Distress Syndrome. Am J Respir Crit Care Med. 2006;173(8):882–9. doi:10.1164/rccm.200508-1196OC

36. Liu KD, Levitt J, Zhuo H, Kallet RH, Brady S, Steingrub J, et al. Randomized Clinical Trial of Activated Protein C for the Treatment of Acute Lung Injury. Am J Respir Crit Care Med. 2008;178(6):618–23. doi:10.1164/rccm.200803-419OC

37. Rice TW, Mogan S, Hays MA, Bernard GR, Jensen GL, Wheeler AP. Randomized Trial of Initial Trophic versus Full-Energy Enteral Nutrition in Mechanically Ventilated Patients with Acute Respiratory Failure. Crit Care Med. 2011;39(5):967–74. doi:10.1097/CCM.0b013e31820a905a

38. Matthay MA, Brower RG, Carson S, Douglas IS, Eisner M, Hite D, et al. Randomized, Placebo-controlled Clinical Trial of an Aerosolized β2-Agonist for Treatment of Acute Lung Injury. Am J Respir Crit Care Med. 2011;184(5):561–8. doi:10.1164/rccm.201012-2090OC

39. Bennett-Guerrero E, Romeiser JL, Talbot LR, Ahmed T, Mamone LJ, Singh SM, et al. Severe Acute Respiratory Syndrome Coronavirus 2 Convalescent Plasma Versus Standard Plasma in Coronavirus Disease 2019 Infected Hospitalized Patients in New York: A Double-Blind Randomized Trial. Crit Care Med. 2021;49(7):1015–25. doi:10.1097/CCM.0000000000005066

40. McAuley DF, Laffey JG, O’Kane CM, Perkins GD, Mullan B, Trinder TJ, et al. Simvastatin in the Acute Respiratory Distress Syndrome. N Engl J Med. 2014;371(18):1695–703. doi:10.1056/NEJMoa1403285

41. Sterne JAC, Savović J, Page MJ, Elbers RG, Blencowe NS, Boutron I, et al. RoB 2: a revised tool for assessing risk of bias in randomised trials. BMJ. 2019;366:l4898. doi:10.1136/bmj.l4898

42. Page MJ, McKenzie JE, Bossuyt PM, Boutron I, Hoffmann TC, Mulrow CD, et al. The PRISMA 2020 statement: an updated guideline for reporting systematic reviews. BMJ. 2021;372:n71. doi:10.1136/bmj.n71

43. Haddaway NR, Page MJ, Pritchard CC, McGuinness LA. PRISMA2020: An R package and Shiny app for producing PRISMA 2020-compliant flow diagrams, with interactivity for optimised digital transparency and Open Synthesis. Campbell Syst Rev. 2022;18(2):e1230. doi:10.1002/cl2.1230

Tables

Table S1. Characteristics of the included studies (for each study).

| Authors | Title | Year^*^ | Journal | Group – ratio (E/C)^†^ | Study type | Center^‡^ | Interim analysis^§^ | Early stop^ll^ | Population | Overall RoB2^¶^ |
| --- | --- | --- | --- | --- | --- | --- | --- | --- | --- | --- |
| Morris et al. | A double-blind placebo-controlled study to evaluate the safety and efficacy of L-2-oxothiazolidine-4-carboxylic acid in the treatment of patients with acute respiratory distress syndrome.[15] | 2008 | CCM | 2 – 1:1 | Sup | Multi. | Yes | Yes | ARDS |  |
| Paine et al. | A randomized Trial of recombinant human granulocyte-macrophage colony stimulating factor for patients with acute lung injury.[16] | 2012 | CCM | 2 – 1:1 | Sup | Multi. | No | Yes | ALI or ARDS |  |
| Mackle et al. | Conservative oxygen therapy during mechanical ventilation in the ICU.[17] | 2020 | NEJM | 2 – 1:1 | Sup | Multi. | Yes | No | MV |  |
| Villar et al. | Dexamethasone treatment for the acute respiratory distress syndrome: a multicentre, randomised controlled trial.[18] | 2020 | LRM | 2 – 1:1 | Sup | Multi. | Yes | Yes | ARDS |  |
| Zhou et al. | Early application of airway pressure release ventilation may reduce the duration of mechanical ventilation in acute respiratory distress syndrome.[19] | 2017 | ICM | 2 – 1:1 | Sup | Mono. | No | No | ARDS |  |
| Trouillet et al. | Early percutaneous tracheotomy versus prolonged intubation of mechanically ventilated patients after cardiac surgery: A Randomized Trial.[20] | 2011 | AIM | 2 – 1:1 | Sup | Mono. | No | No | Cardiac surgery + MV |  |
| Simonis et al. | Effect of a Low vs Intermediate Tidal Volume Strategy on Ventilator-Free Days in Intensive Care Unit Patients Without ARDS: A Randomized Clinical Trial.[21] | 2018 | JAMA | 2 – 1:1 | Sup | Multi. | No | No | No ARDS + MV |  |
| Algera et al. | Effect of a lower vs higher positive end-expiratory pressure strategy on ventilator-free days in ICU patients without ARDS: A randomized clinical trial.[22] | 2020 | JAMA | 2 – 1:1 | N-Inf | Multi. | No | No | No ARDS + MV |  |
| Tomazini et al. | Effect of Dexamethasone on Days Alive and Ventilator-Free in Patients with Moderate or Severe Acute Respiratory Distress Syndrome and COVID-19: The CoDEX Randomized Clinical Trial.[23] | 2020 | JAMA | 2 – 1:1 | Sup | Multi. | Yes | Yes | ARDS + COVID-19 |  |
| Grieco et al. | Effect of Helmet Noninvasive Ventilation vs High-Flow Nasal Oxygen on Days Free of Respiratory Support in Patients with COVID-19 and Moderate to Severe Hypoxemic Respiratory Failure: The HENIVOT Randomized Clinical Trial.[24] | 2021 | JAMA | 2 – 1:1 | Sup | Multi. | No | No | ARF + COVID-19 |  |
| van Meenen et al. | Effect of On-Demand vs Routine Nebulization of Acetylcysteine With Salbutamol on Ventilator-Free Days in Intensive Care Unit Patients Receiving Invasive Ventilation: A Randomized Clinical Trial.[25] | 2018 | JAMA | 2 – 1:1 | N-Inf | Multi. | Yes | No | MV |  |
| Spragg et al. | Effect of recombinant surfactant protein C-based surfactant on the acute respiratory distress syndrome.[26] | 2004 | NEJM | 2 – 1:1 | Sup | Multi. | No | No | ARDS |  |
| Welte et al. | Efficacy and safety of trimodulin, a novel polyclonal antibody preparation, in patients with severe community-acquired pneumonia: a randomized, placebo-controlled, double-blind, multicenter, phase II trial (CIGMA study).[27] | 2018 | ICM | 2 – 1:1 | Sup | Multi. | Yes | No | Community-acquired pneumonia |  |
| Rice et al. | Enteral omega-3 fatty acid, γ-linolenic acid, and antioxidant supplementation in acute lung injury.[28] | 2011 | JAMA | 2 – 1:1 | Sup | Multi. | Yes | Yes | ALI |  |
| Bernard et al. | Evaluating the efficacy and safety of two doses of the polyclonal anti-tumor necrosis factor-α Fragment antibody AZD9773 in Adult patients with severe sepsis and/or septic shock: Randomized, double-blind, placebo-controlled phase IIb Study.[29] | 2014 | CCM | 3 – 1:1:1 | Sup | Multi. | No | No | Sepsis |  |
| Chung et al. | High-frequency percussive ventilation and low tidal volume ventilation in burns: A randomized controlled trial.[30] | 2010 | CCM | 2 – 1:1 | Sup | Mono. | Yes | Yes | Burns |  |
| Rice et al. | Initial trophic vs full enteral feeding in patients with acute lung injury: The EDEN randomized trial.[31] | 2012 | JAMA | 2 – 1:1 | Sup | Multi. | Yes | No | ALI |  |
| Bein et al. | Lower tidal volume strategy (≈3 ml/kg) combined with extracorporeal CO2 removal versus 'conventional' protective ventilation (6 ml/kg) in severe ARDS: the prospective randomized Xtravent-study.[32] | 2013 | ICM | 2 – 1:1 | Sup | Multi. | Yes | Yes | ARDS |  |
| Hodgson et al. | Maximal Recruitment Open Lung Ventilation in Acute Respiratory Distress Syndrome (PHARLAP). A Phase II, Multicenter Randomized Controlled Clinical Trial.[33] | 2019 | AJRCCM | 2 – 1:1 | Sup | Multi. | Yes | Yes | ARDS |  |
| Kacmarek et al. | Neurally adjusted ventilatory assist in acute respiratory failure: a randomized controlled trial.[34] | 2020 | ICM | 2 – 1:1 | Sup | Multi. | No | No | ARF |  |
| Kacmarek et al. | Partial liquid ventilation in adult patients with acute respiratory distress syndrome.[35] | 2006 | AJRCCM | 3 – 1:1:1 | Sup | Multi. | Yes | No | ARDS |  |
| Liu et al. | Randomized clinical trial of activated protein C for the treatment of acute lung injury.[36] | 2008 | AJRCCM | 2 – 1:1 | Sup | Multi. | Yes | No | ALI |  |
| Rice et al. | Randomized trial of initial trophic versus full-energy enteral nutrition in mechanically ventilated patients with acute respiratory failure.[37] | 2011 | CCM | 2 – 1:1 | Sup | Multi. | Yes | No | ARF |  |
| Matthay et al. | Randomized, placebo-controlled clinical trial of an aerosolized β₂-agonist for treatment of acute lung injury.[38] | 2011 | AJRCCM | 2 – 1:1 | Sup | Multi. | Yes | Yes | ALI |  |
| Bennett-Guerrero et al. | Severe Acute Respiratory Syndrome Coronavirus 2 Convalescent Plasma Versus Standard Plasma in Coronavirus Disease 2019 Infected Hospitalized Patients in New York: A Double-Blind Randomized Trial.[39] | 2021 | CCM | 2 – 4:1 | Sup | Mono. | No | Yes | COVID-19 |  |
| McAuley et al. | Simvastatin in the acute respiratory distress syndrome.[40] | 2014 | NEJM | 2 – 1:1 | Sup | Multi. | Yes | No | ARDS |  |

*AIM* Annals of internal medicine, *AJRCCM* American Journal of Respiratory and Critical Care Medicine, *ALI* acute lung injury *ARDS* acute respiratory distress syndrome, *ARF* acute respiratory failure, *CCM* Critical Care Medicine, *ICM* Intensive Care Medicine, *ICU* intensive care unit, *JAMA* Journal of the American Medical Association, *LRM* The Lancet Respiratory Medicine, *MV* mechanical ventilation, *NEJM* The New England Journal of Medicine, *N-Inf* noninferiority, *Sup* superiority. **^*^**The year of publication.
†The number of group and the allocation ratio (E for experimental group and C for control group).
‡If the study is multicentric (multi.) or monocentric (mono.).
**^§^**If there are an interim analysis.
**^ll^**If the study stopped earlier than expected.
**^¶^**The overall risk-of-bias via RoB2 (Revised Cochrane risk-of-bias tools for randomized trials)[41]: low risk; some concerns; high risk.

Table S2. Risk-of-bias summary: review authors’ judgments about each risk-of-bias item based on Revised Cochrane risk-of-bias tools for randomized trials (RoB2)[41].

| Study | D1 | D2 | D3 | D4 | D5 | Overall |
| --- | --- | --- | --- | --- | --- | --- |
| Morris et al., 2008[15] |  |  |  |  |  |  |
| Paine et al., 2012[16] |  |  |  |  |  |  |
| Mackle et al., 2020[17] |  |  |  |  |  |  |
| Villar et al., 2020[18] |  |  |  |  |  |  |
| Zhou et al., 2017[19] |  |  |  |  |  |  |
| Trouillet et al., 2011[20] |  |  |  |  |  |  |
| Simonis et al., 2018[21] |  |  |  |  |  |  |
| Algera et al., 2020[22] |  |  |  |  |  |  |
| Tomazini et al., 2020[23] |  |  |  |  |  |  |
| Grieco et al., 2021[24] |  |  |  |  |  |  |
| van Meenen et al., 2018[25] |  |  |  |  |  |  |
| Spragg et al., 2004[26] |  |  |  |  |  |  |
| Welte et al., 2018[27] |  |  |  |  |  |  |
| Rice et al., 2011[28] |  |  |  |  |  |  |
| Bernard et al., 2014[29] |  |  |  |  |  |  |
| Chung et al., 2010[30] |  |  |  |  |  |  |
| Rice et al., 2012[31] |  |  |  |  |  |  |
| Bein et al., 2013[32] |  |  |  |  |  |  |
| Hodgson et al., 2019[33] |  |  |  |  |  |  |
| Kacmarek et al., 2020[34] |  |  |  |  |  |  |
| Kacmarek et al., 2006[35] |  |  |  |  |  |  |
| Liu et al., 2008[36] |  |  |  |  |  |  |
| Rice et al., 2011[37] |  |  |  |  |  |  |
| Matthay et al., 2011[38] |  |  |  |  |  |  |
| Bennett-Guerrero et al., 2021[39] |  |  |  |  |  |  |
| McAuley et al., 2014[40] |  |  |  |  |  |  |

*D* domain, *D1* randomization process, *D2* Deviations from the intended interventions, *D3* Missing outcome data, *D4* Measurement of the outcome, *D5* Selection of the reported result; low risk; some concerns; high risk.

Table S3. Parameters reported for the sample size estimation of ventilator-free days (for each study).

| Study | VFDs (Ctrl. / Exp. group)^*^ | Mean diff. ± SD^†^ | VFDs distribution / Correction?^‡^ | Statistical model | $\boldsymbol{\alpha}$ risk / Power (%) | Tail(s) | Dropout rate (%) | Mortality considered^§^ |
| --- | --- | --- | --- | --- | --- | --- | --- | --- |
| Morris et al., 2008[15] | NA / NA | NA ± NA | NA / NA | ANOVA | 5 / NA | NA | 0 | No |
| Paine et al., 2012[16] | NA / NA | 3.9 ± NA | Asymmetric / No | Wilcoxon | 5 / 80 | Two | 0 | No |
| Mackle et al., 2020[17] | 16.4 / 19 | 2.6 ± 11.3 | Not normal / + 15% | Quantile regression | 5 / 90 | Two | 1 | No |
| Villar et al., 2020[18] | 9 / 11 | 2 ± 10.7 | Asymmetric / Markov chain | Wilcoxon | 5 / 80 | Two | 0 | Yes |
| Zhou et al., 2017[19] | 14.5 / 19.5 | 5 ± 10.4 | Not normal / No | Kruskal-Wallis | 5 / 80 | Two | 25 | No |
| Trouillet et al., 2011[20] | 23 / 30 | 7 ± 17 | NA / NA | NA | 5 / 80 | Two | 9 | No |
| Simonis et al., 2018[21] | 23 / 24 | 1 ± 5 | NA / NA | Student | 5 / 80 | Two | 20 | No |
| Algera et al., 2020[22] | 16 / 16 | 1.6± 10 | ZI 𝛃 / Spe. model | GAMLSS | 5 / 80 | One | 10 | No |
| Tomazini et al., 2020[23] | 8 / 11 | 3 ± 9 | ZI 𝛃 binomial / Spe. model & + 15% | GLM | 5 / 80 | Two | 4 | No |
| Grieco et al., 2021[24] | 11.6 / 14.5 | 2.9 ± 5 | Normal / NA | NA | 5 / 80 | Two | 10 | No |
| van Meenen et al., 2018[25] | NA / NA | 0.5 ± NA | NA / NA | Wilcoxon | 5 / 80 | One | 5 | No |
| Spragg et al., 2004[26] | NA / NA | 4 ± 10 | Asymmetric / Nonparam. test | Cox | 5 / 80 | Two | 0 | No |
| Welte et al., 2018[27] | NA / NA | 2 ± 3 | Normal / NA | Wilcoxon | 2.5 / 80 | One | 20 | No |
| Rice et al., 2011[28] | 14 / 16.25 | 2.25 ± 10.5 | NA / NA | ANOVA | 5 / 90.7 | Two | 0 | No |
| Bernard et al., 2014[29] | NA / NA | 4.2 ± NA | Not normal / Nonparam. test | ANOVA | 10 / 90 | One | 0 | No |
| Chung et al., 2010[30] | 13 / 15 | 2 ± 7 | NA / NA | NA | 5 / 80 | Two | 0 | No |
| Rice et al., 2012[31] | 14 / 16.25 | 2.25 ± 10.5 | NA / NA | ANOVA | 4.29 / 90.7 | Two | 0 | No |
| Bein et al., 2013[32] | 6 / 11 | 5 ± 10 | NA / NA | NA | 5 / 80 | Two | 10 | No |
| Hodgson et al., 2019[33] | NA / NA | NA ± NA | Not normal / + 15% | NA | 5 / 80 | Two | 5 | No |
| Kacmarek et al., 2020[34] | 21 / 23 | 2 ± 6 | NA / NA | Wilcoxon & Bootstrap | 5 / 80 | Two | 0 | No |
| Kacmarek et al., 2006[35] | NA / NA | 4 ± NA | NA / NA | GLM | 5 / 80 | Two | 0 | No |
| Liu et al., 2008[36] | 12 / 18.5 | 6.5 ± 11 | Not normal / Nonparam. test | Wilcoxon | 5 / 80 | Two | 0 | No |
| Rice et al., 2011[37] | 17.6 / 20.6 | 3 ± 7.3 | NA / NA | Wilcoxon | 5 / 80 | Two | 5 | No |
| Matthay et al., 2011[38] | NA / NA | 2.25 ± 10.5 | NA / NA | ANOVA | 5 / 90.7 | Two | 0 | No |
| Bennett-Guerrero et al., 2021[39] | NA / NA | 2.5 ± 6 | NA / NA | Wilcoxon | 5 / 90 | Two | 0 | No |
| McAuley et al., 2014[40] | 12.7 / 15.3 | 2.6 ± 10.6 | Bimodal / Median comparison | Student & Bootstrap | 5 / 80 | Two | 3 | No |

*ANOVA* analysis of variance, *GAMLSS* generalized additive model for location scale and shape, *GLM* generalized linear model, *NA* not available, *Nonparam*. nonparametric, *Spe.* specific, *ZI* zero-inflated.
^*^The expected ventilator-free days (VFDs) in the two groups (Experimental (Exp.) and Control (Ctrl.)).
^†^The expected mean difference and the related standard deviation.
^‡^If the distribution is not normal, was there a correction?
^§^If the mortality is considered or not for the sample size estimation.

Table S4. Sample size estimation according to statistical test analysis, based on expected parameters and effect size.

| Study | SS estimated | SS Student | SS Wilcoxon | SS Noether | SS Cox | SS ZINB | SS F&G | Effect size |
| --- | --- | --- | --- | --- | --- | --- | --- | --- |
| Morris et al., 2008[15] | 352 | NA | NA | NA | NA | NA | NA | Absolute mean difference |
| Paine et al., 2012[16] | 200 | NA | NA | NA | NA | NA | NA | Absolute difference |
| Mackle et al., 2020[17] | 1000 | 796 | 915 | 1273 | 1582 | 1524 | NA | Absolute mean difference |
| Villar et al., 2020[18] | 320 | 901 | 1036 | 1441 | 614 | 1054 | NA | Absolute difference |
| Zhou et al., 2017[19] | 138 | 138 | 158 | 218 | 282 | 296 | NA | Absolute mean difference |
| Trouillet et al., 2011[20] | 216 | 187 | 215 | 297 | 351 | 130 | NA | Absolute mean difference |
| Simonis et al., 2018[21] | 952 | 787 | 905 | 1258 | 13653 | 10448 | NA | Absolute difference |
| Algera et al., 2020[22] | 980 | 483 | 556 | 1549 | 2228 | 1823 | NA | Absolute mean difference / Mean ratio |
| Tomazini et al., 2020[23] | 350 | 285 | 327 | 453 | 244 | 446 | NA | Absolute mean difference |
| Grieco et al., 2021[24] | 110 | 95 | 110 | 150 | 497 | 663 | NA | Relative mean difference |
| van Meenen et al., 2018[25] | 950 | NA | NA | NA | NA | NA | NA | Absolute median difference |
| Spragg et al., 2004[26] | 440 | 198 | 228 | 315 | NA | NA | NA | Absolute mean difference |
| Welte et al., 2018[27] | 160 | 73 | 84 | 113 | NA | NA | NA | Absolute difference |
| Rice et al., 2011[28] | 1000 | 941 | 1082 | 1505 | 1586 | 1765 | NA | Absolute mean difference |
| Bernard et al., 2014[29] | 300 | NA | NA | NA | NA | NA | NA | Absolute mean difference |
| Chung et al., 2010[30] | 170 | 387 | 445 | 297 | 1208 | 1499 | NA | Absolute mean difference |
| Rice et al., 2012[31] | 1000 | 978 | 1125 | 1565 | 1665 | 1835 | NA | Absolute mean difference |
| Bein et al., 2013[32] | 120 | 128 | 147 | 201 | 67 | 146 | NA | Absolute mean difference |
| Hodgson et al., 2019[33] | 340 | NA | NA | NA | NA | NA | NA | Relative SD difference |
| Kacmarek et al., 2020[34] | 306 | 285 | 327 | 453 | 2988 | 2432 | NA | Absolute mean difference |
| Kacmarek et al., 2006[35] | 309 | NA | NA | NA | NA | NA | NA | Absolute mean difference |
| Liu et al., 2008[36] | 90 | 92 | 106 | 144 | 132 | 157 | NA | Absolute mean difference |
| Rice et al., 2011[37] | 200 | 188 | 216 | 298 | 998 | 928 | NA | Relative difference |
| Matthay et al., 2011[38] | 1000 | 941 | 1082 | 1505 | NA | NA | NA | Absolute mean difference |
| Bennett-Guerrero et al., 2021[39] | 500 | 363 | 418 | 606 | NA | NA | NA | Absolute mean difference |
| McAuley et al., 2014[40] | 540 | 524 | 602 | 837 | 713 | 888 | NA | Absolute mean difference |

*F&G* Fine and Gray regression, *NA* not available, *SD* standard deviation, *SS* sample size, *ZINB* zero-inflated negative binomial regression.

Table S5. Observed ventilator-free days and mortality rate according to group.

| Study | VFDs ctrl. group | Ctrl. group | VFDs exp. group | Exp. group | Mean diff. ± SD | Mortality frame | Mort rate ctrl. group | Mort rate exp. group | Dropout rate (%) |
| --- | --- | --- | --- | --- | --- | --- | --- | --- | --- |
| Morris et al., 2008[15] | 13.5 | Placebo | 8.3 | OTZ | 5.2 ± 10.2 | 28 | 15.8 | 29.7 | 0.5 |
| Paine et al., 2012[16] | 10.7 | Placebo | 10.8 | GM-CSF | 0.1 ± 10.4 | 28 / 180 | 23 / 27 | 17 / 22 | 1.5 |
| Mackle et al., 2020[17] | 16 | Conservative O_2_ | 15.5 | Usual O_2_ | 0.5 ± 11.7 | 90 / 180 | 32.5 / 34.5 | 34.7 / 35.7 | 0 |
| Villar et al., 2020[18] | 7.5 | Standard care | 12.3 | Standard care + Dexamethasone | 4.8 ± 9.5 | 60 | 36 | 21 | 0 |
| Zhou et al., 2017[19] | 5.7 | LTV | 16.3 | APRV | 10.6 ± 11.0 | ICU / hosp. | 34.3 / 37.3 | 19.7 / 23.9 | 0 |
| Trouillet et al., 2011[20] | 28.3 | Prolonged ventilation | 30.4 | Early tracheotomy | 2.1 ± 23.1 | 28 / 60 / 90 | 21 / 28 / 30 | 16 / 26 / 30 | 0 |
| Simonis et al., 2018[21] | 15.5 | Intermediate tidal volume | 15.2 | Low tidal volume | 0.3 ± 11.5 | 28 / 90 | 32.1 / 37.8 | 34.9 / 39.1 | 0.6 |
| Algera et al., 2020[22] | 14.4 | Higher PEEP | 14.8 | Lower PEEP | 0.4 ± 19.8 | 28 / 90 | 42 / 44.3 | 38.4 / 41.6 | 1.1 |
| Tomazini et al., 2020[23] | 4 | Standard care | 6.6 | Standard care + Dexamethasone | 2.6 ± 9.1 | 28 | 61.5 | 56.3 | 0 |
| Grieco et al., 2021[24] | 13 | HFNO | 15 | Helmet NIV | 2 ± 11.0 | 28 / 60 | 18 / 22 | 15 / 24 | 0.9 |
| van Meenen et al., 2018[25] | 15.3 | Routine nebulization | 15.6 | On demand nebulization | 0.3 ± 19.3 | 28 / 90 | 31.9 / 36.7 | 31 / 38.5 | 2.3 |
| Spragg et al., 2004[26] | 9 | Standard care | 8.2 | Standard care + surfactant | 0.8 ± 15.8 | 28 | 32 | 36 | 0 |
| Welte et al., 2018[27] | 9.6 | Placebo | 11 | Trimodulin | 1.4 ± 9.5 | 28 | 27.8 | 22.2 | 0 |
| Rice et al., 2011[28] | 17.2 | Control | 14 | n-3 supplement | 3.2 ± 10.7 | 28 | 17.6 | 25.1 | 0 |
| Bernard et al., 2014[29] | 18.3 | Placebo | 19.7 | AZD9773 (low or high dose) | 1.4 ± 15.2 | 28 | 20 | 15 | 1.3 |
| Chung et al., 2010[30] | 11 | LTV | 12 | HFPV | 1 ± 9.0 | 28 | 19 | 19 | 0 |
| Rice et al., 2012[31] | 15 | Full feeding | 14.9 | Trophic feeding | 0.1 ± 10.9 | 60 | 22.2 | 23.2 | 0 |
| Bein et al., 2013[32] | 9.3 | Conventional tidal | 10 | Lower tidal + avECCO_2_-R | 0.7 ± 8.5 | hosp. | 15.4 | 17.5 | 0 |
| Hodgson et al., 2019[33] | 12 | Protective ventilation | 12.3 | Max lung recruitment | 0.3 ± 16.2 | 28 / 90 / 180 | 26.8 / 30.4 / 30.4 | 24.6 / 24.6 / 24.6 | 1.7 |
| Kacmarek et al., 2020[34] | 14 | Conventional ventilation | 16.7 | NAVA | 2.7 ± 17.3 | 90 | 32.7 | 26.8 | 1.3 |
| Kacmarek et al., 2006[35] | 13 | Conventional ventilation | 7.4 | PLV (low or high dose) | 5.6 ± 8.9 | 28 | 15 | 26.3 | 0 |
| Liu et al., 2008[36] | 14.3 | Placebo | 18.3 | Activated protein C | 4 ± 13.8 | 60 | 13.5 | 13.5 | 0 |
| Rice et al., 2011[37] | 17.8 | Full feeding | 17.9 | Trophic feeding | 0.1 ± 10.5 | 28 | 19.6 | 22.4 | 0 |
| Matthay et al., 2011[38] | 16.6 | Placebo | 14.4 | Albuterol | 2.2 ± 0.9 | 60 / 90 | 17.7 / 18.5 | 23 / 24.3 | 0 |
| Bennett-Guerrero et al., 2021[39] | 18.7 | Standard plasma | 19.3 | Convalescent plasma | 0.6 ± 21.4 | 28 / 90 | 27 / 33 | 24 / 27 | 0 |
| McAuley et al., 2014[40] | 11.5 | Placebo | 12.6 | Simvastatin | 1.1 ± 10.2 | 28 | 26.8 | 22 | 0.6 |

*APRV* airway pressure-release ventilation, *HFNO* high-flow nasal oxygen, *HFPV* high-frequency percussive ventilation, *hosp*. hospital, *ICU* intensive care unit, *LTV* low-tidal volume ventilation, *NA* not available, *NAVA* neurally-adjusted ventilation assist, *NIV* noninvasive ventilation, *OTZ* oxothiazolidine-4-carboxylic acid, *PEEP* positive end-expiratory pressure, *PLV* partial liquid ventilation, *SD* standard deviation, *VFDs* ventilator-free days.

Table S6. Sample size estimation according to statistical test analysis, based on observed parameters, and author’s conclusion.

| Study | SS observed | Mean difference | SS Student | SS Wilcoxon | SS Noether | SS Cox | SS ZINB | Authors’ conclusion |
| --- | --- | --- | --- | --- | --- | --- | --- | --- |
| Morris et al., 2008[15] | 215 | 5.2 | 123 | 141 | 194 | 105 | 165 | Significant difference |
| Villar et al., 2020[18] | 277 | 4.8 | 124 | 143 | 196 | 101 | 184 | Significant difference |
| Zhou et al., 2017[19] | 138 | 10.6 | 36 | 41 | 54 | 22 | 45 | Significant difference |
| Trouillet et al., 2011[20] | 216 | 2.1 | 3787 | 4355 | 6068 | 4827 | 1624 | No difference between groups |
| Tomazini et al., 2020[23] | 299 | 2.6 | 383 | 440 | 611 | 99 | 329 | Significant difference |
| Grieco et al., 2021[24] | 110 | 2 | 952 | 1094 | 1522 | 1208 | 1499 | No difference between groups |
| Welte et al., 2018[27] | 160 | 1.4 | 1432 | 1647 | 2293 | 1334 | 2215 | No difference between groups |
| Rice et al., 2011[28] | 272 | 3.2 | 480 | 552 | 767 | 831 | 903 | Significant difference |
| Bernard et al., 2014[29] | 300 | 1.4 | 1877 | 2159 | 4933 | 4977 | 5314 | No difference between groups |
| Chung et al., 2010[30] | 62 | 1 | 2545 | 2927 | 4077 | 3267 | 4868 | No difference between groups |
| Kacmarek et al., 2020[34] | 310 | 2.7 | 1286 | 1479 | 2058 | 795 | 907 | Significant difference |
| Kacmarek et al., 2006[35] | 311 | 5.6 | 151 | 174 | 191 | 117 | 210 | Significant difference |
| Liu et al., 2008[36] | 75 | 4 | 375 | 432 | 599 | 407 | 442 | No difference between groups |
| Matthay et al., 2011[38] | 282 | 2.2 | 10 | 11 | 14 | 1742 | 1894 | No difference between groups |
| McAuley et al., 2014[40] | 540 | 1.1 | 2677 | 3078 | 4288 | 2964 | 4226 | No difference between groups |

*NA* not available, *SS* sample size, *ZINB* zero-inflated negative binomial regression.
We did not include the sample size estimation based on the Fine and Gray regression because the extubation incidences were missing. Moreover, we did not estimate the sample size when the mean difference was too low (i.e., when the mean difference was less than 1). Indeed, conducting a study with such an effect size would appear irrelevant and clinically unrealistic.

Table S7. Sample size estimation from the simulation of Bodet-Contentin et al.[12] according to the statistical test analysis.

|  | Ctrl. Group | Exp. group |
| --- | --- | --- |
| $\boldsymbol{\alpha}$risk (%) | 5 | |
| Power (%) | 80 | |
| VFDs | 10.8 | 10.4 |
| Mean difference | 0.46 | |
| Standard deviation | 10.8 | 10.2 |
| Extubation rate | 0.572 | 0.596 |
| Mortality rate | 0.28 | 0.15 |
| SS Student | 16519 | |
| SS Wilcoxon | 18997 | |
| SS Noether | 26478 | |
| SS Cox | 13339 | |
| SS ZINB | 21437 | |
| SS FG | 1777 | |

*ctrl.* control, *exp*. experimental, *F&G* Fine and Gray, *SS* sample size, *VFDs* ventilator-free days, *ZINB* zero-inflated negative binomial regression. However, it is more of a thought experiment because the effect size was low (mean difference = 0.46).

Table S8. PRISMA 2020 Article Checklist[42].

| **Topic** | **No.** | **Item** | **Location where item is reported** |
| --- | --- | --- | --- |
| **TITLE** |  |  |  |
| **Title** | 1 | Identify the report as a systematic review. | Title page |
| **ABSTRACT** |  |  |  |
| **Abstract** | 2 | See the PRISMA 2020 for Abstracts checklist |  |
| **INTRODUCTION** |  |  |  |
| **Rationale** | 3 | Describe the rationale for the review in the context of existing knowledge. | Page 3 |
| **Objectives** | 4 | Provide an explicit statement of the objective(s) or question(s) the review addresses. | Page 4 |
| **METHODS** |  |  |  |
| **Eligibility criteria** | 5 | Specify the inclusion and exclusion criteria for the review and how studies were grouped for the syntheses. | Page 4-5 |
| **Information sources** | 6 | Specify all databases, registers, websites, organisations, reference lists and other sources searched or consulted to identify studies. Specify the date when each source was last searched or consulted. | Page 4 |
| **Search strategy** | 7 | Present the full search strategies for all databases, registers and websites, including any filters and limits used. | Page 4 + Methods in the additional file 1 |
| **Selection process** | 8 | Specify the methods used to decide whether a study met the inclusion criteria of the review, including how many reviewers screened each record and each report retrieved, whether they worked independently, and if applicable, details of automation tools used in the process. | Page 4 |
| **Data collection process** | 9 | Specify the methods used to collect data from reports, including how many reviewers collected data from each report, whether they worked independently, any processes for obtaining or confirming data from study investigators, and if applicable, details of automation tools used in the process. | Page 5 + Methods in the additional file 1 |
| **Data items** | 10a | List and define all outcomes for which data were sought. Specify whether all results that were compatible with each outcome domain in each study were sought (e.g. for all measures, time points, analyses), and if not, the methods used to decide which results to collect. | Methods in the additional file 1 |
|  | 10b | List and define all other variables for which data were sought (e.g. participant and intervention characteristics, funding sources). Describe any assumptions made about any missing or unclear information. | Methods in the additional file 1 |
| **Study risk of bias assessment** | 11 | Specify the methods used to assess risk of bias in the included studies, including details of the tool(s) used, how many reviewers assessed each study and whether they worked independently, and if applicable, details of automation tools used in the process. | Page 5 |
| **Effect measures** | 12 | Specify for each outcome the effect measure(s) (e.g. risk ratio, mean difference) used in the synthesis or presentation of results. | Methods in the additional file 1 |
| **Synthesis methods** | 13a | Describe the processes used to decide which studies were eligible for each synthesis (e.g. tabulating the study intervention characteristics and comparing against the planned groups for each synthesis (item 5)). | NA |
|  | 13b | Describe any methods required to prepare the data for presentation or synthesis, such as handling of missing summary statistics, or data conversions. | Methods in the additional file 1 |
|  | 13c | Describe any methods used to tabulate or visually display results of individual studies and syntheses. | NA |
|  | 13d | Describe any methods used to synthesize results and provide a rationale for the choice(s). If meta-analysis was performed, describe the model(s), method(s) to identify the presence and extent of statistical heterogeneity, and software package(s) used. | NA |
|  | 13e | Describe any methods used to explore possible causes of heterogeneity among study results (e.g. subgroup analysis, meta-regression). | NA |
|  | 13f | Describe any sensitivity analyses conducted to assess robustness of the synthesized results. | NA |
| **Reporting bias assessment** | 14 | Describe any methods used to assess risk of bias due to missing results in a synthesis (arising from reporting biases). | Page 5 |
| **Certainty assessment** | 15 | Describe any methods used to assess certainty (or confidence) in the body of evidence for an outcome. | NA |
| **RESULTS** |  |  |  |
| **Study selection** | 16a | Describe the results of the search and selection process, from the number of records identified in the search to the number of studies included in the review, ideally using a flow diagram. | Page 6 + figure S1 |
|  | 16b | Cite studies that might appear to meet the inclusion criteria, but which were excluded, and explain why they were excluded. | Page 6 + figure S1 |
| **Study characteristics** | 17 | Cite each included study and present its characteristics. | Page 6 + Table 1 + Table S1 |
| **Risk of bias in studies** | 18 | Present assessments of risk of bias for each included study. | Page 7 + Table S2 |
| **Results of individual studies** | 19 | For all outcomes, present, for each study: (a) summary statistics for each group (where appropriate) and (b) an effect estimate and its precision (e.g. confidence/credible interval), ideally using structured tables or plots. | Pages 7-11 + Tables 2-3 + Figure 1 + Tables S2-S6 |
| **Results of syntheses** | 20a | For each synthesis, briefly summarise the characteristics and risk of bias among contributing studies. | Table 1 + Table S1-S2 |
|  | 20b | Present results of all statistical syntheses conducted. If meta-analysis was done, present for each the summary estimate and its precision (e.g. confidence/credible interval) and measures of statistical heterogeneity. If comparing groups, describe the direction of the effect. | NA |
|  | 20c | Present results of all investigations of possible causes of heterogeneity among study results. | NA |
|  | 20d | Present results of all sensitivity analyses conducted to assess the robustness of the synthesized results. | NA |
| **Reporting biases** | 21 | Present assessments of risk of bias due to missing results (arising from reporting biases) for each synthesis assessed. | Pages 8-9 |
| **Certainty of evidence** | 22 | Present assessments of certainty (or confidence) in the body of evidence for each outcome assessed. | Table S2 |
| **DISCUSSION** |  |  |  |
| **Discussion** | 23a | Provide a general interpretation of the results in the context of other evidence. | Pages 11-14 |
|  | 23b | Discuss any limitations of the evidence included in the review. | Pages 14 |
|  | 23c | Discuss any limitations of the review processes used. | Pages 14 |
|  | 23d | Discuss implications of the results for practice, policy, and future research. | Pages 11-15 |
| **OTHER INFORMATION** |  |  |  |
| **Registration and protocol** | 24a | Provide registration information for the review, including register name and registration number, or state that the review was not registered. | Page 5 |
|  | 24b | Indicate where the review protocol can be accessed, or state that a protocol was not prepared. | Pages 2 & 5 & 19 |
|  | 24c | Describe and explain any amendments to information provided at registration or in the protocol. | NA |
| **Support** | 25 | Describe sources of financial or non-financial support for the review, and the role of the funders or sponsors in the review. | Pages 16-17 |
| **Competing interests** | 26 | Declare any competing interests of review authors. | Page 16 |
| **Availability of data, code and other materials** | 27 | Report which of the following are publicly available and where they can be found: template data collection forms; data extracted from included studies; data used for all analyses; analytic code; any other materials used in the review. | Page 16 |

*PRISMA* Preferred Reporting Items for Systematic Reviews and Meta-Analyses.
For more information, visit: www.prisma-statement.org.

Table S9. PRISMA 2020 Abstract Checklist[42].

| **Topic** | **No.** | **Item** | **Reported?** |
| --- | --- | --- | --- |
| **TITLE** |  |  |  |
| **Title** | 1 | Identify the report as a systematic review. | Yes |
| **BACKGROUND** |  |  |  |
| **Objectives** | 2 | Provide an explicit statement of the main objective(s) or question(s) the review addresses. | Yes |
| **METHODS** |  |  |  |
| **Eligibility criteria** | 3 | Specify the inclusion and exclusion criteria for the review. | Yes |
| **Information sources** | 4 | Specify the information sources (e.g. databases, registers) used to identify studies and the date when each was last searched. | Yes |
| **Risk of bias** | 5 | Specify the methods used to assess risk of bias in the included studies. | Yes |
| **Synthesis of results** | 6 | Specify the methods used to present and synthesize results. | Yes |
| **RESULTS** |  |  |  |
| **Included studies** | 7 | Give the total number of included studies and participants and summarise relevant characteristics of studies. | Yes |
| **Synthesis of results** | 8 | Present results for main outcomes, preferably indicating the number of included studies and participants for each. If meta-analysis was done, report the summary estimate and confidence/credible interval. If comparing groups, indicate the direction of the effect (i.e. which group is favoured). | Yes |
| **DISCUSSION** |  |  |  |
| **Limitations of evidence** | 9 | Provide a brief summary of the limitations of the evidence included in the review (e.g. study risk of bias, inconsistency and imprecision). | Yes |
| **Interpretation** | 10 | Provide a general interpretation of the results and important implications. | Yes |
| **OTHER** |  |  |  |
| **Funding** | 11 | Specify the primary source of funding for the review. | Yes |
| **Registration** | 12 | Provide the register name and registration number. | Yes |

*PRISMA* Preferred Reporting Items for Systematic Reviews and Meta-Analyses.
For more information, visit: www.prisma-statement.org.

Figures


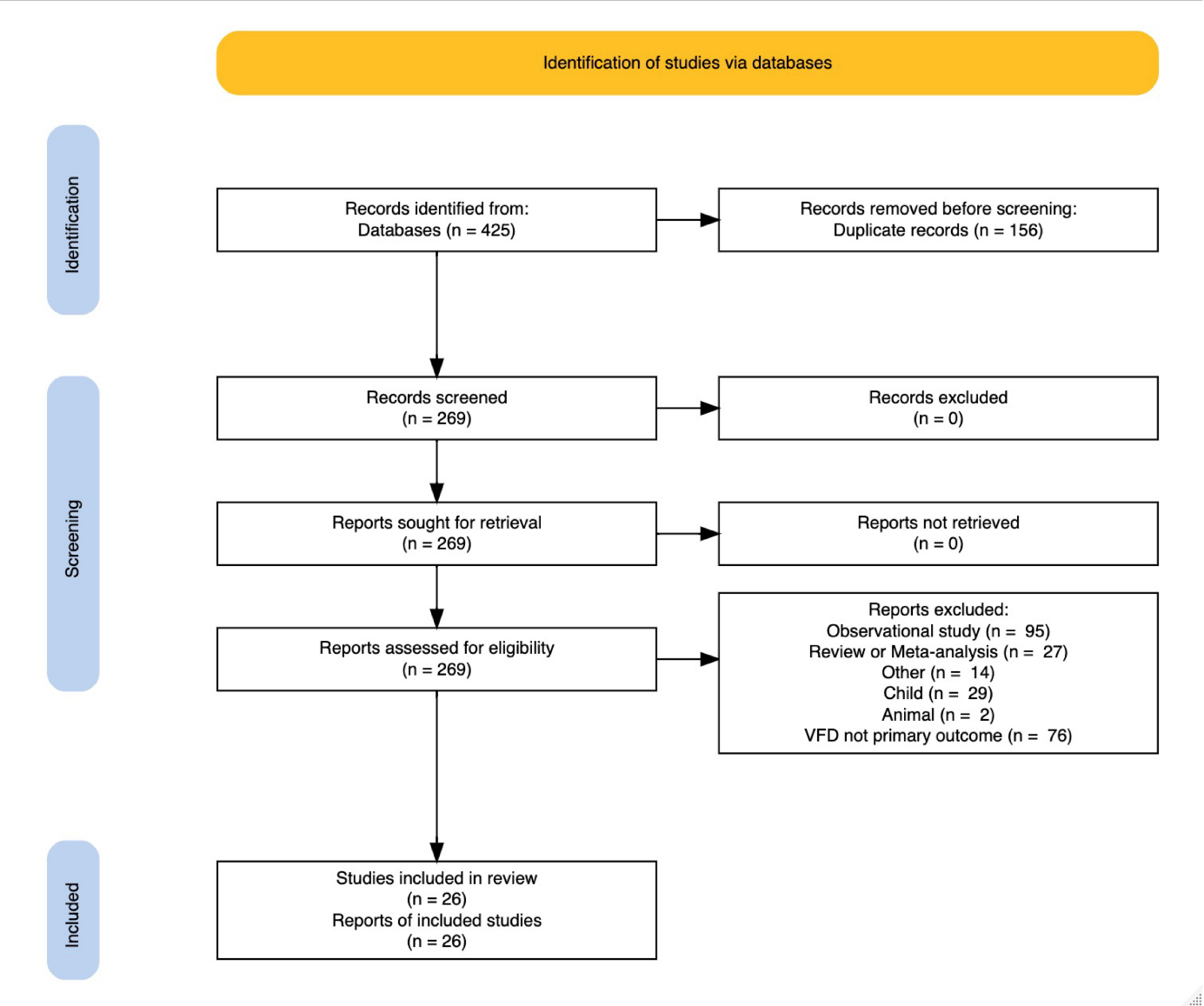
Figure S1. PRISMA[42] flow diagram from search in December 2021.

*PRISMA* Preferred Reporting Items for Systematic Reviews and Meta-Analyses.
This diagram was generated with the R[3] package DiagrammeR[43].

Figure S2. Expected (A) mean difference in ventilator-free days and (B) related standard deviation (B) in the 26 studies included in the systematic review.


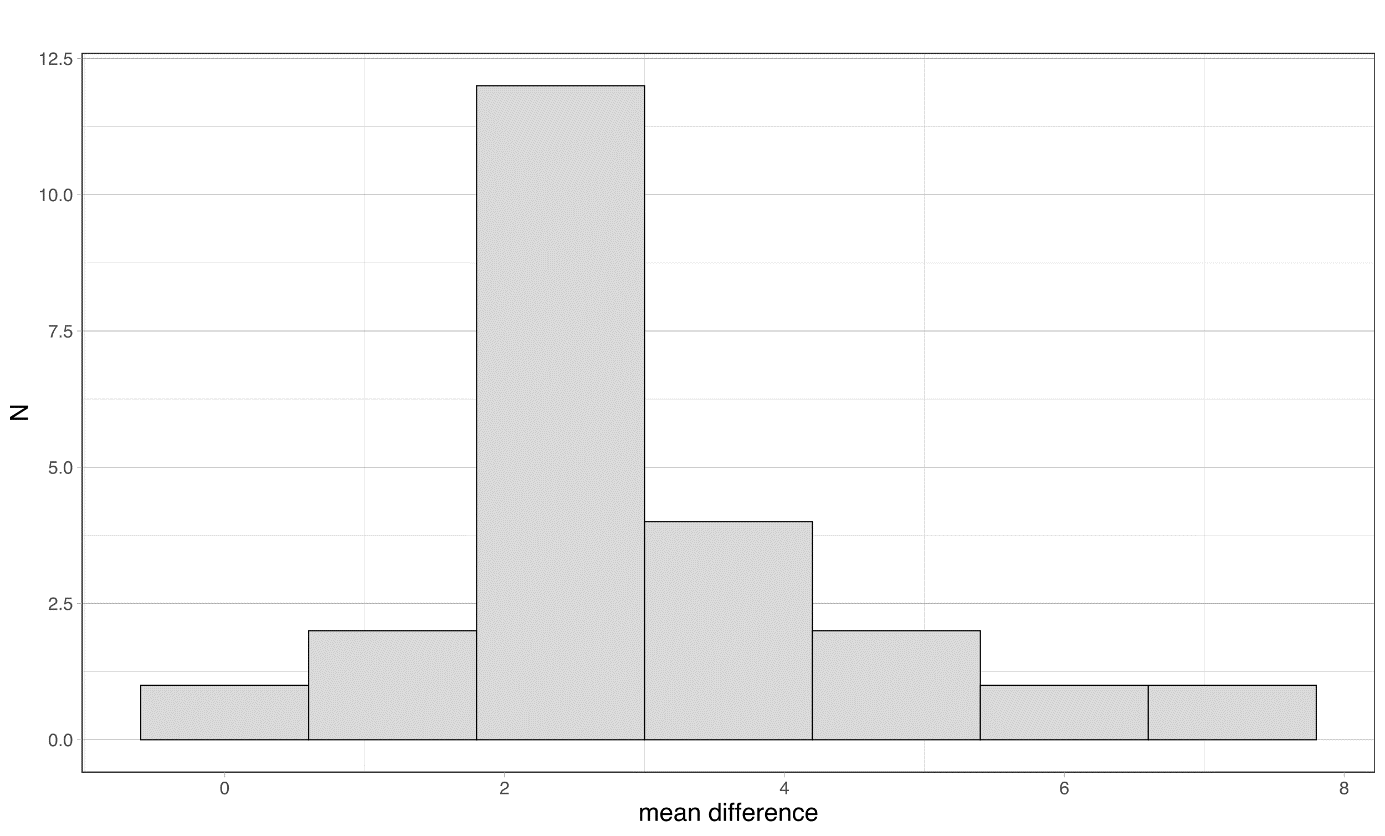

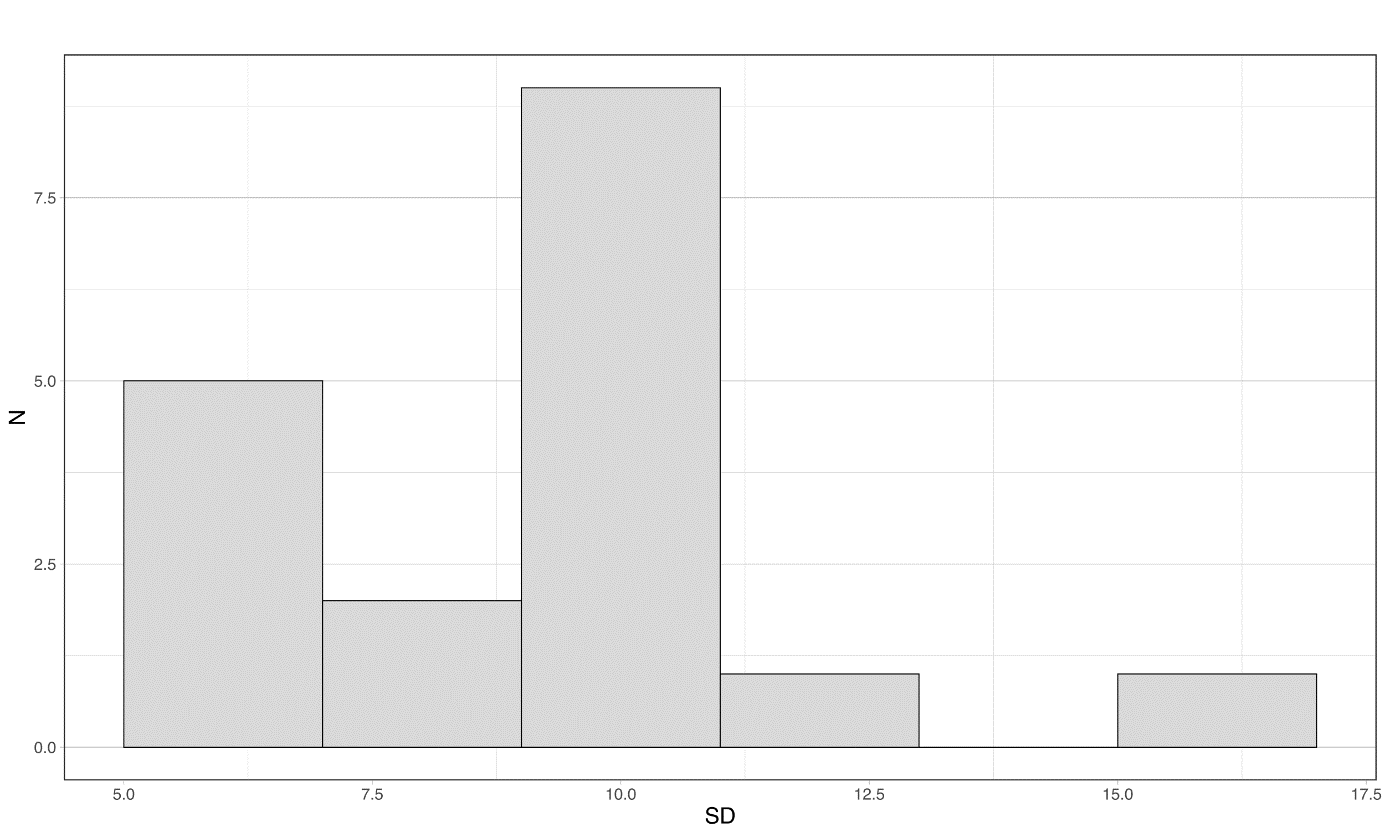


**A**

**B**

*N* number of studies, *SD* standard deviation.
